# *De-Novo* Identification of PPARγ/RXR Binding Sites and Direct Targets during Adipogenesis

**DOI:** 10.1371/journal.pone.0004907

**Published:** 2009-03-20

**Authors:** Mohamed Sabry Hamza, Sebastian Pott, Vinsensius B. Vega, Jane S. Thomsen, Gopalan Srinivasan Kandhadayar, Patrick Wei Pern Ng, Kuo Ping Chiu, Sven Pettersson, Chia Lin Wei, Yijun Ruan, Edison T. Liu

**Affiliations:** 1 Genome Institute of Singapore, Agency for Science, Technology and Research (A*STAR), Singapore, Singapore; 2 Department of Microbiology, Tumor and Cell Biology, Karolinska Institute, Stockholm, Sweden; University of Camerino, Italy

## Abstract

**Background:**

The pathophysiology of obesity and type 2 diabetes mellitus is associated with abnormalities in endocrine signaling in adipose tissue and one of the key signaling affectors operative in these disorders is the nuclear hormone transcription factor peroxisome proliferator-activated receptor-γ (PPARγ). PPARγ has pleiotropic functions affecting a wide range of fundamental biological processes including the regulation of genes that modulate insulin sensitivity, adipocyte differentiation, inflammation and atherosclerosis. To date, only a limited number of direct targets for PPARγ have been identified through research using the well established pre-adipogenic cell line, 3T3-L1. In order to obtain a genome-wide view of PPARγ binding sites, we applied the pair end-tagging technology (ChIP-PET) to map PPARγ binding sites in 3T3-L1 preadipocyte cells.

**Methodology/Principal Findings:**

Coupling gene expression profile analysis with ChIP-PET, we identified in a genome-wide manner over 7700 DNA binding sites of the transcription factor PPARγ and its heterodimeric partner RXR during the course of adipocyte differentiation. Our validation studies prove that the identified sites are bona fide binding sites for both PPARγ and RXR and that they are functionally capable of driving PPARγ specific transcription. Our results strongly indicate that PPARγ is the predominant heterodimerization partner for RXR during late stages of adipocyte differentiation. Additionally, we find that PPARγ/RXR association is enriched within the proximity of the 5′ region of the transcription start site and this association is significantly associated with transcriptional up-regulation of genes involved in fatty acid and lipid metabolism confirming the role of PPARγ as the master transcriptional regulator of adipogenesis. Evolutionary conservation analysis of these binding sites is greater when adjacent to up-regulated genes than down-regulated genes, suggesting the primordial function of PPARγ/RXR is in the induction of genes. Our functional validations resulted in identifying novel PPARγ direct targets that have not been previously reported to promote adipogenic differentiation.

**Conclusions/Significance:**

We have identified in a genome-wide manner the binding sites of PPARγ and RXR during the course of adipogenic differentiation in 3T3L1 cells, and provide an important resource for the study of PPARγ function in the context of adipocyte differentiation.

## Introduction

The pathophysiology of obesity and type 2 diabetes mellitus is associated with abnormalities in endocrine signaling in adipose tissue and one of the key signaling affectors operative in these disorders is the nuclear hormone transcription factor peroxisome proliferator-activated receptor-γ (PPARγ) [1]–[4]. PPARγ has pleiotropic functions affecting a wide range of fundamental biological processes including the regulation of genes that modulate insulin sensitivity, adipocyte differentiation, inflammation and atherosclerosis. Recent evidence has also implicated PPARγ in cell cycle control and cancer progression [5]–[9]. PPARγ is activated by several naturally occurring compounds, and synthetic molecules, such as thiazolidinediones, which are actively used in the therapy of type 2 diabetes [8], [10]–[12]. To date, only a limited number of direct targets for PPARγ have been identified through research using the well established pre-adipogenic cell line, 3T3-L1.

Recently, *in silico* analysis of the human genome sequence has been carried out to identify direct target genes of PPARγ [13]. In addition, a model for PPARγ binding preferences using position weight matrices derived from all published Peroxisome Proliferator Response Elements (PPREs) has been reported [14]. Upon activation, PPARγ heterodimerizes with the retinoid X receptor (RXR) and transcriptionally regulates target genes by binding to response elements (PPRE) consisting of a hexameric DNA core recognition motif spaced by one nucleotide (DR-1, PPREs; 5′-AGGTCANAGGTCA-3′), where PPARγ occupies the 5′-half of the motif [15], [16]. However, *in silico* predictions poorly correlate with *in vivo* transcription factor binding, and as such, this approach has limited ability to predict *bona fide* PPARγ binding.

To better define the transcriptional control functions of PPARγ and its heterodimerization partner, RXR, during adipogenesis, we employed the paired-end ditag (PET) technology to characterize chromatin immunoprecipitated (ChIP)-enriched DNA fragments and mapped, in an unbiased manner, the binding sites of these nuclear hormone receptors (NHR) on a genome-wide scale. Previous work from our group has shown the utility of this approach in assessing transcription factor control mechanisms, and the potential advantages of sequenced based methodologies over hybridization approaches in mapping transcription factor binding sites [17]. Combining the ChIP-PET identification of PPARγ and of RXR binding sites with PPARγ dependent gene expression analysis of cells undergoing adipogenesis, we have comprehensively assessed their binding site usage and the regulatory mechanisms governing the transcriptional control of direct target genes. Our genomic scale data suggests that RXR is the major heterodimeric partner for PPARγ during adipocyte differentiation and maturation. Additionally, we find that PPARγ/RXR association is enriched within the proximity of the 5′ region of the transcription start site and is significantly associated with transcriptional up-regulation of genes confirming the role of PPARγ as the master transcriptional regulator of adipogenesis. Interestingly, we observed that during the early stages of adipocyte differentiation (fibroblasts to preadipocytes), the proportion of repressed genes were higher to those of genes that were induced. This relation was reversed after day 3, suggesting a concomitant with the switch from early to terminal adipocyte differentiation and marked by the induction of adipogenic maintenance genes.

Our validation studies prove that the identified sites are bona fide binding sites and that they are transcriptionally capable of driving PPARγ specific transcription. In addition, we have functionally assessed PPARγ/RXR target genes and deduced the metabolic pathways that they activate during the course of differentiation.

## Results

### Utilizing 3T3-L1 cells to investigate PPARγ dependent regulatory networks during adipogenesis

**Figure 1:**
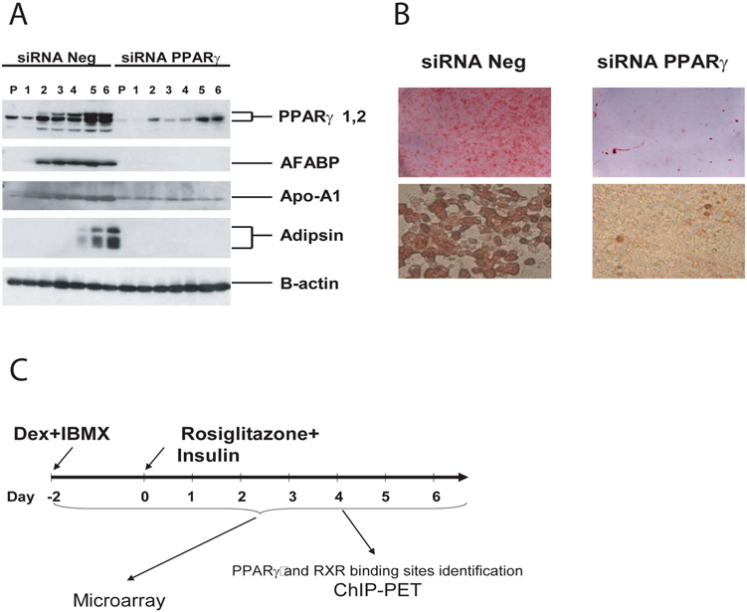
3T3L1 cells as model system for adipocyte differentiation. Undifferentiated 3T3-L1 fibroblast undergoes adipogenesis upon treatment with Dexamethasone and IBMX, followed by Insulin and Rosiglitazone after two days. Adipogenesis was completely abrogated by PPARγ specific siRNA. a) Adipogenesis was monitored by Western blot for induction of adipocyte-specific marker genes. Beta-actin served as loading control. b) accumulation of lipid droplets in differentiated 3T3-L1 cells was visualized by OilRed-O staining c) Schematic outline of the experimental setup: 3T3-L1 cell were treated with Dexamethasone and IBMX at confluency 2 days before stimulation with Rosiglitazone and Insulin. Samples for ChIP were taken at day 4. Samples for expression analysis were taken at all time points indicated. For each time point cells treated with PPARγ specific and control siRNA prior to the differentiation protocol were collected in 3 biological replicates. Microarray data was submitted to the GEO repository under GEO Submission (GSE12929).

We used the well established 3T3-L1 cell line as a model system for adipocyte differentiation. Following a protocol schematically outlined in Figure 1C, 3T3-L1 cells were differentiated into adipocytes 4 days post treatment with dexamethasone and IBMX, followed by insulin and rosiglitazone after two days. Differentiation was verified both by western blot for adipocyte specific markers and accumulation of lipids within the cells as detected by Oil-Red O staining. 3T3-L1 cell transfected with PPARγ specific siRNA prior to differentiation completely abolished this process (Figures 1 A, B).

### Genes differentially expressed by PPARγ siRNA during adipogenesis provide the first stratum in constructing a PPARγ dependent regulatory network

We followed gene expression changes during adipogenesis of 3T3-L1 cells treated with PPARγ-specific siRNA (PPARγ knock down (KD)) and those treated with control non-targeting siRNA using Illumina Sentrix BeadhipsRef-8. RNA was extracted at each timepoint (everyday). Analysis using all time-points (days −2 to 6) revealed that 1845 probes (correspond to about 1700 genes) were differentially expressed between PPARγ KD and control siRNA (Spreadsheet S1). After hierarchical clustering, two distinct expression patterns were observed with approximately 40% down and 60% up-regulated in the PPARγ KD. Henceforth, we will refer to genes down-regulated and up-regulated in the PPARγ KD as induced and repressed by PPARγ respectively. Analyzing the expression profile day 4–6 separately, however, provided us with 760 probes differentially expressed with the majority of genes induced by PPARγ (Spreadsheet S2), 222 probes of which were not present in the set including all time-points. The global expression profile changed over the course of differentiation. During the early stages of adipocyte differentiation, the proportion of PPARγ repressed genes (difference to control >1.5 fold) was higher than that of genes induced by PPARγ (Figure S1 A). This relation was reversed at day 3, concomitant with the switch from early to terminal adipocyte differentiation and marked by the induction of adipogenic maintenance genes. Gene ontology analysis for the combined set (day (−2 to 6) and (4 to 6); 1656 genes with annotation) of differentially expressed genes using PANTHER [18], [19] showed an enrichment of genes involved in lipid, fatty acid and steroid metabolism, amino acid metabolism and generally genes with metabolic function. Only PPARγ induced genes were significantly associated with metabolic processes while PPARγ repressed genes were enriched in processes associated with development and cell division (Figure S1 B).

### Global map of PPARγ and RXR binding sites

Having identified genes whose expression is PPARγ dependent during adipogenesis, we aimed to further uncover genes directly regulated by PPARγ through mapping PPARγ binding sites on a genome-wide scale. As RXR has been shown to be a heterodimerization partner of PPARγ [20], we also independently mapped RXR direct binding sites. We posited that the overlay of the RXR sites onto the PPARγ sites could potentially improve the identification of functional PPARγ binding sites. In addition, from our previous work, the digital count of ditags at binding loci is well correlated with the transcription factor occupancy. Thus, the combined analysis of PPARγ and RXR binding ditag counts at close locations may be an indication of the importance of specific PPARγ∶RXR interactions during PPARγ induced differentiation at a genome-wide level.

In order to identify binding sites, we collected chromatin immunopreciptation (ChIP) material from differentiated 3T3-L1 cells using PPARγ and RXR specific antibodies. To maximize ChIP enrichment, the samples were harvested on day 6 post induction, when PPARγ levels were found to be highest during the differentiation process (Figure 1A). PPARγ and RXR bound DNA fragments were isolated from 3T3-L1 cells. Immunoprecipitated DNA fragments were sequenced using the pair-end-tagging approach and mapped to reference genome (mm8) [21], [22]. 3,211,429 of clone equivalents (ditags) were sequenced from the PPARγ ChIP-PET library and 1,646,083 ditags were sequenced from the RXR library. Both libraries reached approximately 63–65% sequencing saturation (*see* saturation analysis in supplementary material, Figure S2).

**Figure 2:**
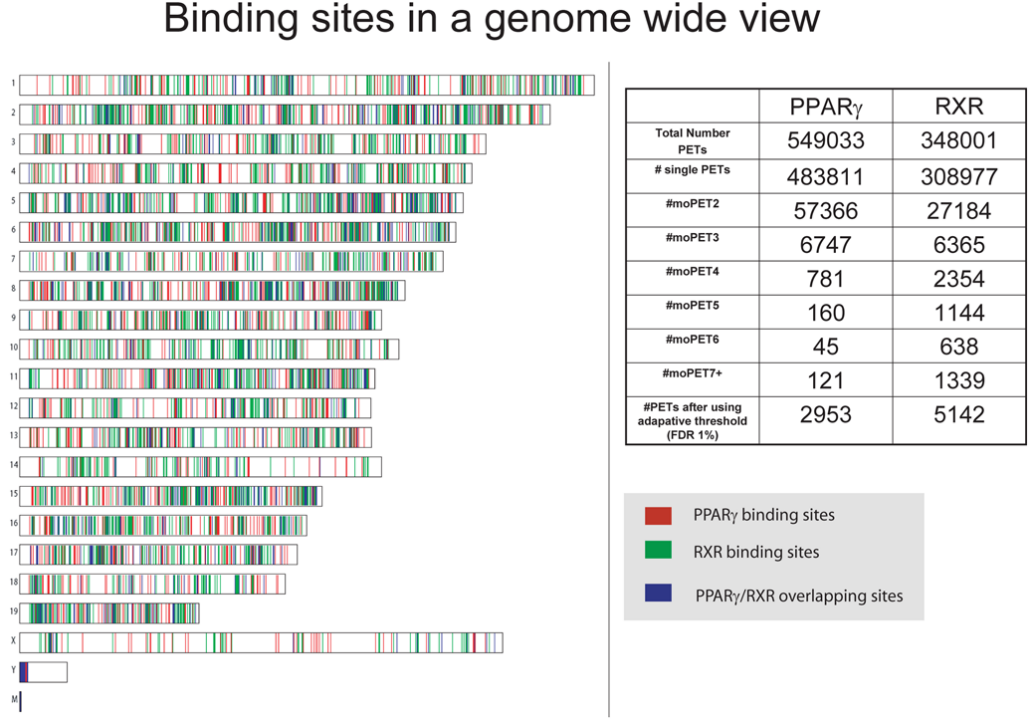
Binding regions of PPARγ and RXR are abundant but not unevenly dispersed. Genome-wide view of PPARγ and RXR binding regions, binned into 200 kbp blocks. No particular chromosomal concentration of bins containing PPARγ only (red), RXR only (green), or both (blue) was observed. Summary of the moPET counts for the PPARγ and RXR ChIP-PET libraries. Good binding regions were identified using aberration normalizing algorithm [25].

Overlapping PET fragments were clustered as before [23], [24] to form ChIP-PET clusters. The resultant ChIP-PET clusters were then stratified based on the maximum number of overlapping fragments within each cluster. For example, a non-overlapping fragment is labeled as a moPET1 cluster, and as before, these are considered background/noise [21], while a moPET3 cluster is one where the maximum number of PET fragments overlapping at any location within the cluster is three. (moPET*n*+ clusters is the set of moPET*i* clusters, where *i*≥*n*). Applying the adaptive thresholding method [25], 2953 PPARγ ChIP-PET clusters and 5142 RXR ChIP-PET clusters were found to be statistically significant (FDR of ≤0.01) and henceforth referred to as putative binding regions (Figure 2).

**Figure 3:**
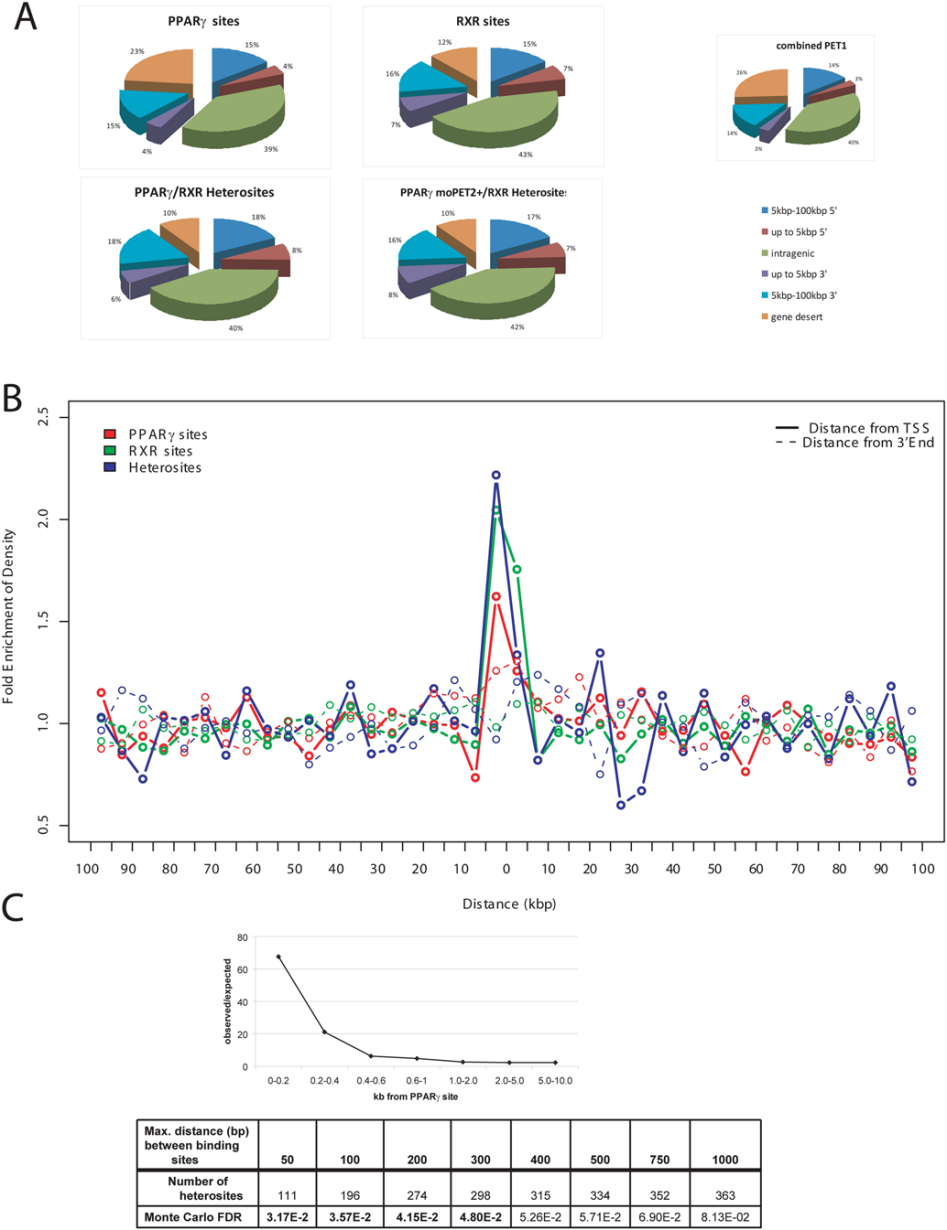
PPARγ and RXR binding regions are similarly distributed, with respect to nearest transcription unit. (A) Proportion of PPARγ and RXR binding regions' locations relative to the nearest transcription units. PPARγ monosites (no RXR sites within 200 bp), RXR monosites (no PPARγ sites within 200 bp), high confidence PPARγ∶RXR heterosites (within 200 bp of each other) and low confidence PPARγmoPET2∶RXR heterosites (within 200 bp of each other). Pie Chart on the right shows the distribution of the combined Pet1 fragments from both the PPARγ and the RXR libraries on the same genomic regions. Distribution of Pet1 fragments is assumed to be random (or close to) and was therefore chosen as background comparison to visualize biases in the binding site distribution in the PPARγ and RXR libraries. (B) PPARγ binding regions, RXR binding regions, as well as commonPPARγ∶RXR binding regions are biased towards 5′ ends of known transcription units. Density of binding sites around TSS and 3 prime ends of known genes (+/−100 kb) is plotted for PPARγ, RXR, and conjoint PPARγ∶RXR binding sites. Coordinates for TSS and 3 prime ends of known genes were extracted from UCSC KnownGenes database. C) Distribution of PPARγ and RXR binding sites indicates interaction of PPARγ and RXR. Number of RXR binding sites in proximity to PPARγ sites was determined. Plot of the ration of observed number of RXR sites within the distance window indicated over the number of sites expected using randomly distributed genomic region. Monte-Carlo estimated FDR of PPARγ∶RXR co-occurrences for various maximum distances between PPARγ and RXR binding sites.

Similar to other nuclear hormone receptors such as the estrogen receptor, the majority (75–88%) of the bona fide binding regions of PPARγ and RXR are within genes or within 100 kb of their 5′ and 3′end; with the plurality being within the genes (39–43%) (Figure 3 A). The binding regions showed no obvious concentration in any specific genomic area when mapped to genome. Genomic distribution of PPARγ moPET1 and RXR moPET1 fragments were used to approximate background distribution (Figure S11).

Such a coarse analysis, however, will not reveal potentially significantly but less frequent positional clustering of binding sites. To address this possibility, we assessed whether PPARγ or RXR would bind at a frequency greater than chance vis-à-vis specific gene boundaries such as the transcriptional start site (TSS). We found that the proportion of binding sites of either NHR within 5 kb of the TSS was significantly higher than downstream of a gene. When we mapped the frequency contours for PPARγ and RXR binding within 100 kbp 5′ and 3′ of TSS, we found a distinct enrichment within approximately 5–10 kb of the TSS (Figure 3B). No such bias was observed when the 3′ prime end of genes was assessed.

### Heterodimerization of PPARγ and RXR are evident in the binding sites proximity

**Figure 4:**
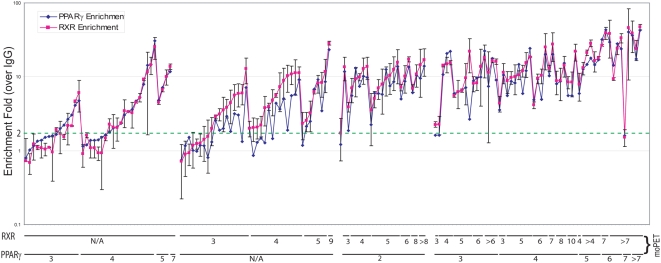
PPARγ∶ RXR overlap sites show higher validation rate than nonoverlapping sites. ChIP Q-PCR was used for validation of binding sites. 4 populations of binding sites were chosen for validation: PPARγ monosites (no RXR sites within 200 bp), RXR monosites (no PPARγ sites within 200 bp), low confidence PPARγmoPET2∶RXR heterosites (within 200 bp) and high confidence PPARγ∶RXR heterosites (within 200 bp). High confidence PPARγ∶RXR heterosites shows, on average, higher ChIP-qPCR enrichment than PPARγ or RXR monosites and PPARγmoPET2∶RXR heterosites. Shown is the enrichment over an unspecific antibody control. Each data point represents mean value of 3 biological replicates, with error bars indicating the standard deviation.

The heterodimerization between PPARγ and RXR has been previously determined on a small number of individual binding sites and is thought to be the exclusive biochemical configuration for transcriptional activation by PPARγ [26], [27]. Our data allowed us to examine this issue at a genome-wide scale. Taking the middle of ChIP-PET overlap region (i.e., a PET cluster) as the proxy for actual binding site locations, as in [28], we plotted the ratio of the observed distance distribution between binding sites of PPARγ and RXR (Figure 3C) and what would be expected in case of random binding. We observed a significant co-localization of PPARγ and RXR binding sites within distances of 500 bp or less. A Monte-Carlo assessment further confirmed that the enrichment of paired PPARγ/RXR binding sites is significant at FDR<0.05 when these bona fide individual binding sites are separated by <300 bp (Figure 3C). We call these PPARγ/RXR site-pairs identified by our ChIP-PET sequencing as ChIP-PET *heterosites*, as they are the likely DNA interaction sites of putative PPARγ∶RXR heterodimers (Henceforth, we would also use the term monosites to refer to binding sites of PPARγ (or RXR) where no RXR (or PPARγ) present within the specified distance).The interval size is most likely determined by the number of fragments constituting a cluster and the initial shear size of the DNA fragments. As such, we considered a maximum interval of 200 bp in identifying heterosites. Individual NHR binding sites without another binding cluster within 200 bp are called monosites. Validations of these PPARγ and RXR binding at the monosites and heterosites by ChIP-qPCR (Figure 4) found that heterosites had a significantly greater degree of occupancy than the monosites. Furthermore, this degree of occupancy for any individual site was not observed in undifferentiated cells (fibroblasts), where RXR is abundantly expressed but PPARγ is not (Figure S3). We then took 157 ChIP-PET determined mono- and heterosites and subjected them to ChIP-q-PCR validation for binding of PPARγ and RXR (Figure 4). The results of this validation first showed that on average, the moPET counts correlated with the qPCR outcomes: the higher the moPET, the greater the fold enrichment by qPCR. The second observation is that almost all of the monosites as determined by ChIP-PET for PPARγ were also bound by RXR in qPCR validation. The reverse was also true in that almost all ChIP-PET based monosites for RXR also bound PPARγ by qPCR. This is supported by the fold enrichment levels of PPARγ or RXR at any binding site that exceeded the adaptive threshold cutoff. Regardless of whether monosites or heterosites are used, both NHR bound with the same relative degree to each binding site (Table S4).What discrepancies were attributable to a small number of low order binding sites of approximately 1.5–2 fold enrichment where occupancy of the heterodimer partner could not be detected. Given that each of the ChIP-PET sequencing runs were only 63–65% saturated (Figure S2), we believe that the heterosite calls missed by ChIP-PET is a function of sampling error of sites with weaker NHR occupancy. Highly enriched sites are most likely present in both libraries and sites with lower enrichment are more likely to be present in only one of the libraries. Supporting this, we found that the ChIP-PET monosites had a lower level of enrichment for PPARγ and RXR as compared to the ChIP-PET heterosites upon ChIP-qPCR validation (3.5–5.4 average fold enrichment for the monosites vs. 14.4–16.1 average fold enrichment for the strong heterosites;Wilcoxon's one-tailed test p<1E-4 in all cases) (Table S4). The combination of our genome-wide ChIP-PET data and the ChIP qPCR validation therefore suggest that PPARγ and RXR interact on the same DNA binding sites throughout the genome though of differing intensities. This is consistent with the biochemical evidence that PPARγ and RXR act together as a heterodimer in PPARγ action. Our work extends this observation on a genome wide scale. Combining both the PPARγ and RXR sites we identified a total of 7821 binding sites throughout the genome (PPARγ/RXR heterosites are counted as one) (Figure 2 and Spreadsheets S3, S4 and S5).

These sites therefore represent a substantial part of the genome-wide pool of PPARγ /RXR binding sites operative during adipogenesis. Based on the validation rate we conclude that sites with higher occupancy are more likely to be present in this pool.

Advancing a major aim of this study of identifying direct PPARγ/RXR target genes, we sought to further combine PPARγ dependent expression profiles with the genome-wide PPARγ∶RXR binding site data. We extracted 278 probes on the Illumina expression array which had PPARγmoPET2+/RXR ChIP-PET heterosite(s) within 5 kb of the TSS, and 424 and 1494 probes had respectively a PPARγ or RXR site within 5 kb of their TSS (Spreadsheets S3, S4 and S5). Based on our earlier analysis, the ChIP-PET determined monosites would collectively represent sites with a lower level of occupancy by the heterodimer than the ChIP-PET heterosites. Of the 278 probes in proximity to a heterosite, more than 31% (88) were regulated by PPARγ activation (P = 1.91E-38). This compares to 14% (54) in proximity to PPARγ ChIP-PET monosites (P = 2.57E-07), 13% (192) for RXR ChIP-PET monosites (P = 2.61E-16), and 9% for all probes on the expression array (i.e., background). More specifically, probes induced by PPARγ are more commonly associated with RXR/PPARγ binding sites in proximity of the TSS, whereas there is no association seen with genes repressed by RXR/PPARγ activation (Table 1). Thus, proximity of a binding site with a high level of heterodimer occupancy to a TSS significantly increases the likelihood that an adjacent gene will be up-regulated by PPARγ activation.

**Table 1:** Genes induced by PPARγ are more commonly associated with PPARγ/RXR binding sites in proximity of the TSS.

	# Probes	# of Probes Regulated	# of Probes down-regulated (fraction down) and P-value enrichment	# of Probes up-regulated (fraction up) and P-value enrichment
PPARγ only	424	58	14(3.3%) P = 0.98	44(10.4%) p = 2.57E-07
RXR only	1494	192	54(3.6%) P = 0.99	1384(9.2%) P = 2.61E-16
PPARγ2/RXR	278	88	12(4.3%) P = 0.84	76(27.3%) P = 1.91E-38
Total	20751	2070	1134(5.5%)	936(4.5%)

Proportion of regulated probes with a binding site for PPARγ, RXR, and PPARγmoPET2+RXR is shown. Hypergenometric p-value was calculated comparing the proportion of regulated probes among all probes on the array to the proportion of regulated genes with binding sites within 5 kbp to the proportion of all probes on the array with binding sites within 5 kbp.

In an attempt to improve the identification of true target genes and making use of true PPARγ sites that went undetected (false-negatives), we reasoned that RXR ChIP-PET monosites supported by lower confidence PPARγ binding (as defined by moPET threshold of ≥2) would also identify good PPARγ∶RXR overlap binding sites. Using the RXR monosites to catch PPARγ sites that were not called using the adaptive thresholding methods (false negatives) (Figure S10). Indeed, qPCR validation of RXR monosites that were also identified by a lower threshold moPET2 PPARγ site gave us ∼91% success rate for co-occupancy (Figure 4 S7, S8 and Table S4).

**Figure 5:**
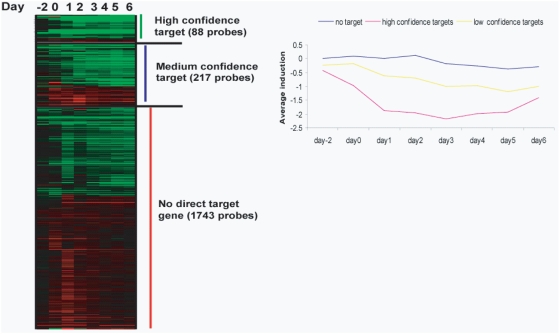
Association of PPARγ∶RXR heterosites with differentially expressed genes. The majority of genes identified as direct PPARγ target genes are induced during adipogenesis. Genes differentially expressed during adipogenesis that are associated with a PPARγ∶RXR heterosite were called high confidence PPARγ target genes. Those with a PPARγ or RXR monosite were labeled as medium confidence targets. Expression profiles of high confidence, medium confidence, and no target genes are shown as heatmaps (day-2-6). Displayed is the fold difference of cells treated with PPARγ specific siRNA over non-targeting siRNA. Values representing the average of 3 biological replicates per conditions. Average fold difference of expression levels between cells treated with PPARγ specific siRNA over non-targeting siRNA was plotted for high confidence, medium confidence, and no target genes was plotted across the timepoints.

To examine this more closely, we collapsed the array probe sets onto genes and classified a gene as target if it is both regulated during adipogenesis and has at least one binding site (PPARγ and RXR) within 5 kb of the gene's TSS. Since it is highly possible that binding sites farther away from the TSS may also regular its proximate gene, these criteria are likely to be more stringent. We identified 75 high confidence target genes as defined by association with heterosites and 180 lower confidence targets defined by association with monosites (Table S2). Further, we assessed their expression dynamics after PPARγ activation (Figure 5). Our analysis revealed that high confidence targets were induced at significantly higher levels than lower confidence target genes. Genes induced by PPARγ are more commonly associated with RXR/PPARγ binding sites in proximity of the TSS (P = 2.57E-07 to P = 1.91E-38) (Table 1). No significant association is seen with genes repressed by PPARγ activation.

### Binding sites show PPARγ-dependent activity in vitro

**Figure 6:**
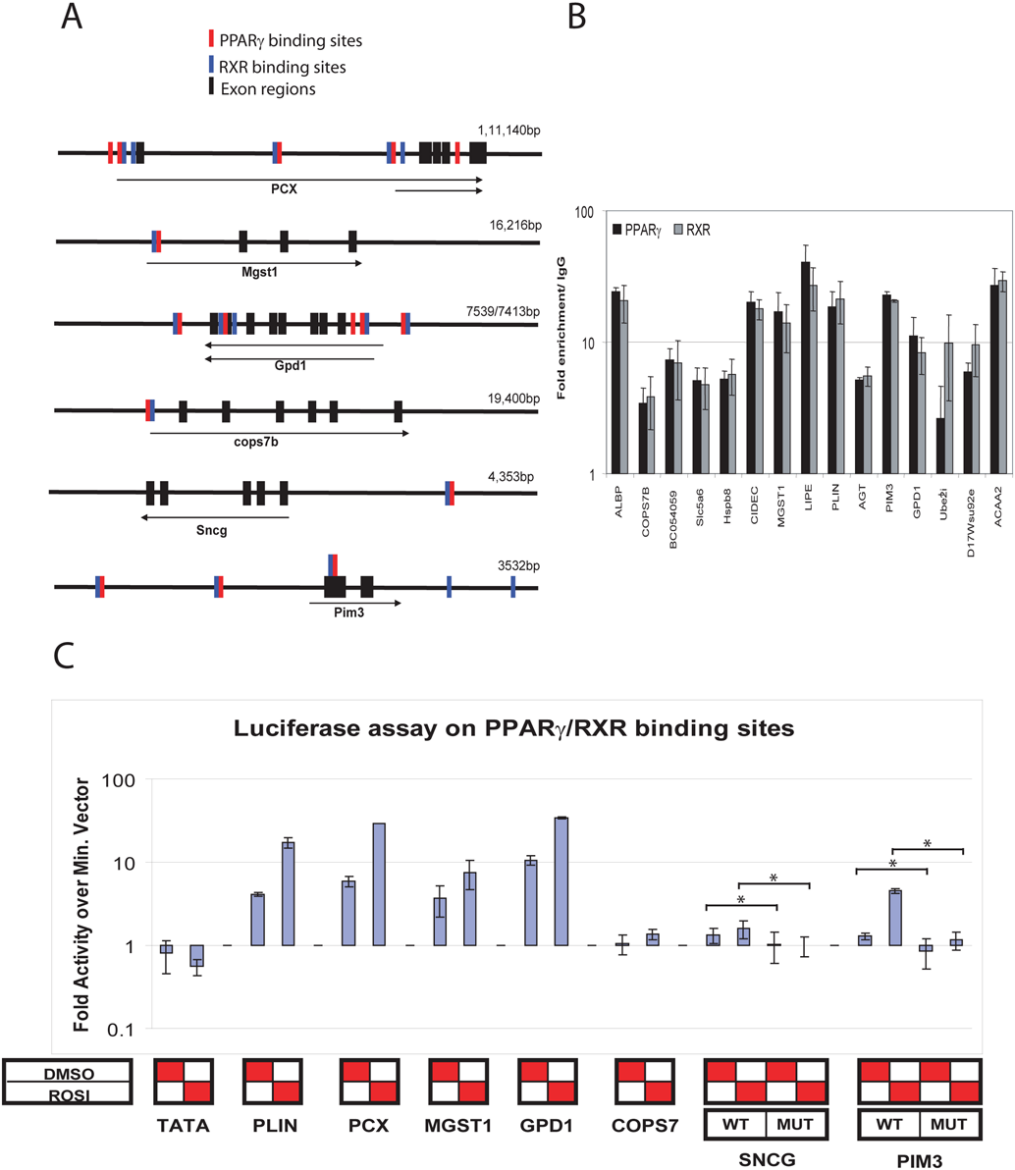
Functional validations of high confidence direct targets. High confidence targets were chosen for further validation to confirm PPARγ responsiveness. A) Localization of PPARγ∶RXR binding sites around putative target genes is shown schematically for genes which were further selected for validation using reporter constructs. B) Binding of PPARγ and RXR was confirmed by ChIP Q-PCR day 4 after adipogenesis induction. Shown is the enrichment of target regions in the PPARγ and RXR specific pull-down over an unspecific antibody control. Values represent the mean of three biological experiments; error bars indicate standard deviations. C) Luciferase assays showing PPARγ dependent activation of luc reporter in 3T3-L1 fibroblasts. Putative PPARγ/RXR binding regions in proximity to Plin, PCX, Mgst1, Gpd1, Cops7b, Sncg and Pim3 were cloned into TATA box containing pGL3 reporter construct. Reporter constructs were co-transfected with PPARγ expression vector or empty vector as control. Putative PPRE in binding region adjacent to Pim3 and SNCG were mutated to confirm functionality. Samples were treated with 1 uM Rosiglitazone or DMSO for 24 h prior to quantization. Luciferase activity was measured and normalized to renilla. Mutation of putative PPARγ/RXR binding motif within the binding region abrogates PPARγ dependent activation of the luciferase reporter construct. Shown is the average of two biological replicates each run in triplicates; with the standard deviations indicated as error bars. Asterisks denote significance (P-value<0.05, student T-test, two-tailed distribution.

To confirm that the PPARγ binding sites identified by ChIP-PET can regulate gene expression, we tested the ability of 6 putative PPARγ DNA binding regions to mediate activation by PPARγ directly with reporter constructs (Figures 6C and S6). These binding regions were selected based on ChIP q-PCR validation and proximity to the TSS (Figure 6 A, B). Enrichment of these binding regions by ChIP q-PCR was only observed in adipocytes when compared with fibroblast (Figure S3). The PPARγ binding regions were cloned into a PGL3 vector with a TATA box [23]. PPARγ binding region from the known target gene PLIN served as a positive control and the empty vector (TATA alone) as the negative control. Reporter constructs were transfected into 3T3-L1 fibroblasts without induction of endogenous PPARγ expression either alone or together with a PPARγ expression plasmid. 24 hrs after transfection, cells were stimulated with 1 uM rosiglitazone or vehicle. Luciferase activity was measure 24 h after stimulation. Four out of the six binding sites tested showed substantial luciferase activity in PPARγ transfected cells over empty vector transfected cells. The two remaining sites tested showed only modest increase in luciferase activity. Further increase in luciferase activity was observed in cells treated with rosiglitazone. We have previously shown that the center of a PET cluster represents the precise position of transcription factor binding site in vivo [23]. By mutating an element which closely resembled the PPARγ/RXR binding consensus motif in one of the binding regions adjacent to the *pim3* and *Sncg* genes (Table S5 and Figure S6), respectively, the reporter gene activation by PPARγ was markedly decreased. This suggests that the binding sites tested can directly induce transcription in a ligand inducible manner and that this induction is likely to be dependent on the specific PPARγ/RXR binding.

### Known PPARγ∶RXR binding motif is overrepresented in the binding regions

Applying *de-novo* motif discovery algorithm, Weeder [29] on the heterosites and further refining the results, akin to what was done in [28], we were able to uncover a weight matrix (A/GGGNCAAAGGTCA) similar to the known weight matrix of PPRE {(A/G)GGTCAAAGGTCA}, a direct hexad repeat that represents PPARγ∶RXR joint binding motif. However, the conspicuous absence of the consensus T in the calculated left repeat and the attenuation of the signal from the T from the right repeat from our genome wide data suggest that the earlier derived consensus may have been a special case. Using the RXR monosites we found a similar structure with two direct repeats, but the T is absent in both sites (AGGGCAAA GGGCA).The PPARγ monosites, however, showed only a signal for a half-site that, though more degenerate, resembles the AGGGCA motif (Figures S4 and S9). In an alternative analysis, we screened the center of the PPARγ and RXR monosites and PPARγmoPET2+/RXR binding regions for the frequency of occurrence of a reported PPARγ/RXR binding motif using previously described PWMs [15], [30], [31]. All categories showed significant enrichment of motif occurrence compared to background genomic regions (Table S3). Comparing consensus motif occurrence in binding regions adjacent to genes down-regulated and up-regulated by PPARγ, we found that repressed genes were less significantly associated with a motif as compared to induced genes (Table S3). Our results show that motif occurrence, like binding site distribution itself, appears to be strongly biased towards regulated genes, more specifically towards genes induced by PPARγ.

### Conservation of PPARγ and RXR sites

Conservation of short sequence sites can be assessed using different metrics. In this study, we examined whether the sequences around the different categories of murine PPARγ and/or RXR binding sites would be conserved in the human. 200 bp interval around the binding sites was chosen to evaluate association with conserved elements (100 bp up- and downstream of the middle of the binding region). We observed that between 29–33% of the PPARγ/RXR heterosites and RXR monosites were conserved between mouse and human whereas PPARγ sites showed little diference from background in terms of conservation (20% and 19% respectively). This low sequence conservation at the binding sites is consistent with our experience with the majority of transcription factors. When we examined the conservation of binding sites within 5 kb of the TSS of PPARγ regulated vs. non-regulated genes, we found a significant enrichment of conserved heterosites with upregulated genes (65% upregulated vs. 42% down regulated, Figure S5). There was no such enrichment with RXR or PPARγ monosites or with RXRPET1 sites which represent background. However, RXR monosites and heterosites of lower quality showed increased association with conserved elements as compared to both RXR and PPARγ∶PET1 clusters. This suggests that a conserved function of PPARγ action is the induction of a number of specific downstream genes.

### PPARγ target genes are significantly associated with metabolic processes

Assuming that direct PPARγ/RXR targets are responsible for a large part of the observed biological effects of PPARγ, we characterized these targets using PANTHER. As before, we considered genes with at least one heterosite or monosite within 5 kb of their TSS a potential target gene. This restrictive classification will leave out a considerable number of true target genes containing a binding site further away than 5 kb, however it will prevent the “dilution” of the true effectors of PPARγ by including a large number of false-positive targets. When all PPARγ and RXR binding sites are taken into consideration, the top functional category that is overrepresented is lipid, fatty acid and steroid metabolism (P = 6.61E-08) followed by nucleic acid metabolism (P = 1.95E-06) and cell proliferation and differentiation (P = 7.02E-05) (Tables S1, S6, S7 and S8). When only the high quality heterosites were considered, lipid, fatty acid and steroid metabolism again was the top category, followed by apoptosis, and then fatty acid metabolism. Gratifyingly, when compared to the ontology of genes regulated by PPARγ as assessed by expression arrays (Figure S1B), the top intersects were lipid, fatty acid and steroid metabolism and fatty acid metabolism. Thus by two different approaches, one based on expression, and the second solely on binding site association, PPARγ primarily regulates a transcriptional cassette that is involved in lipid and fatty acid metabolism.

### siRNA knockdown of a set of PPARγ direct targets affects adipocyte differentiation

**Figure 7:**
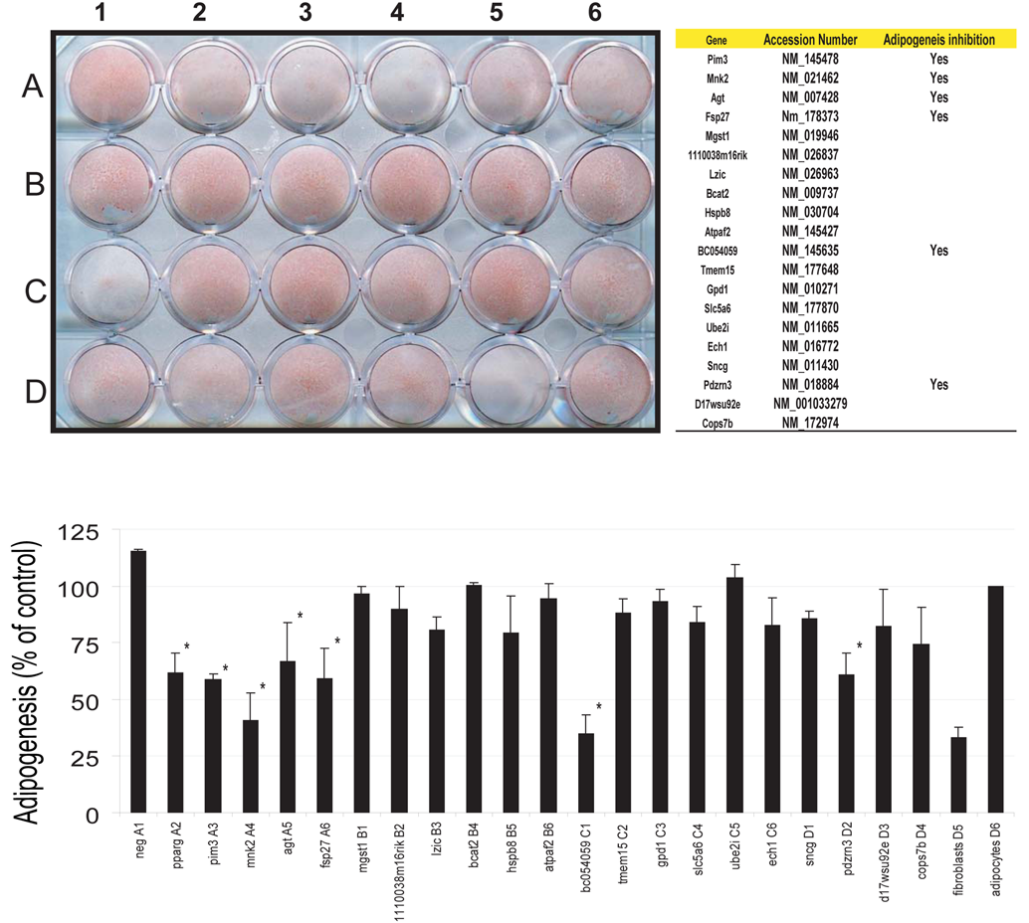
Capacity for adipocyte differentiation after siRNA treatment of selected PPARγ direct target genes. Undifferentiated 3T3-L1 cells were transfected with siRNAs that targeted genes that were selected on the basis of ChIP-PET binding for both PPARγ and RXR, and expression data. Transfected cells were brought to confluence and then treated with IBMX/DEX for 48 hrs, and then stimulated with insulin and rosiglitazone. Cells were stained after 4 days of treatment using Oil Red O stain, and quantified for lipid accumulation using a spectrophometer set at an absorbance of 540 nM. Percentage of adipogenesis, as measured by Oil Red O staining, was compared for each siRNA target to that of the non-transfected differentiated cells (set at 100% adipogenesis). Non-targeting siRNA served as a control, while siRNA targeting PPARγ served as a threshold for adipogenesis inhibition. A gene target was considered affecting lipid accumulation if it did not produce significantly higher lipid accumulation (one-tailed t-test p>0.2) than that of the PPARγ KD. Starred bars represent genes that meet the criteria. Confluent fibroblasts, as well as differentiated 3T3-L1 cells, that were not transfected with siRNA also served as controls to compare the effects of non-targeting siRNA on adipocyte differentiation. Results indicate two biological replicates, each of which was transfected with siRNA to a final concentration of 50 nM.

To assess the function of selected direct PPARγ targets during adipogenesis, we carried out gene knockdown experiments using siRNA in 3T3-L1 cells. Adipogenic potential of the selected target genes was evaluated by quantifying the reduction of lipid accumulation in differentiated adipocytes in the absence of the respective gene. Evaluated genes were selected from the high confidence target genes defined by a transcriptional unit with heterosites within 5 kb of their TSS and showing differential expression pattern during adipogenesis. After Oil Red O staining, lipid accumulation in cells was measured using a spectrometer set at 540 nm. Level of lipid accumulation inhibition ascribed to PPARγ KD was used as the positive control (40% reduction compared with untreated adipocytes). A knockdown was considered affecting lipid accumulation if it did not produce significantly higher lipid accumulation (one-tailed t-test p>0.02) than that of PPARγ KD. The level of lipid accumulation of untransfected cells undergoing adipogenesis after chemical stimulation was taken arbitrarily to exhibit 100% adipogenesis. Whereas non-targeting siRNA had no effect on the adipogenic phenotype, 6 of the 20 (30%) tested siRNAs showed a reduction of lipid accumulation similar to that of PPARγ knockdown. The knockdowns that were biologically effective were *Pim3*, *Mnk2*, *Agt*, *Fsp27*, *Smaf1*(adipogenin) and *Pdzrn3*, while other targets showed no or very modest levels of inhibition of lipid accumulation (Figure 7). These affects on lipogenesis are not due to non specific cellular toxicity (data not shown).

## Discussion

That PPARγ with its heterodimerization partner, RXR, are key regulators of adipogenesis [20], [32]–[34]. Herein, we sought to detail the transcriptional regulatory configuration of PPARγ-RXR-DNA interaction on a genome-wide scale using an established ChIP-PET sequencing approach. Such genome-wide analysis of bona fide NHR binding sites provides sufficient numbers to achieve statistical power to uncover core characteristics of specific transcription factor-DNA interaction by examining the population characteristics of the DNA binding sites in a genome-wide scale. By comprehensively assessing the binding profiles of each heterodimer partner separately and then mapping their intersects, we were able to assess the functional importance of the heterodimer in PPARγ signaling and function.

The observation of consistent PPARγ/RXR interaction at almost all high quality RXR binding sites was unexpected since such exclusive interaction would not be expected given the promiscuous nature of RXR as a heterodimerization partner [35]. In seeking an explanation for these findings, we constructed potential mechanistic models based on the composite configuration of the “population” of binding sites. This approach was used to uncover the cooperation between Oct4 and Sox2 in regulating genes involved in stem cell biology. The Oct4 and Nanog transcription network regulates pluripotency in mouse embryonic stem cells. [28]. Based on our data, we posit that true monosites are fundamentally very weak binders and account for a small proportion of the binding sequences. This is especially true for PPARγ where there were a significantly greater proportion of weak occupancy sites (as measured by the ratio low/high confidence sites) than with RXR (PPARγ = 62783/2953 = 21.3 vs. RXR =  33882/5142 = 6.6).We also observed that experimental heterosites are associated with consistently higher NHR occupancy and more likely associated with genes induced by PPARγ. One plausible model is that our ChIP-PET experiments were performed at day 4 post induction, where PPARγ levels are the highest and in the presence of the potent PPARγ agonist rosiglitazone, thus represents a cellular milieu that favors the full utilization of the PPARγ/RXR heterodimers in adipocyte differentiation. We surmise that, under these conditions, the excess PPARγ could titer out the limited pool of RXR molecules from its other heterodimeric partners during the course of adipogenesis. Consistent with this observation is that despite similar levels of RXR in undifferentiated 3T3L1 cells, the binding of RXR to high confidence sites was uniformly lower than that seen after adipogenic induction when PPARγ expression is dramatically induced (Figure S3). This suggests that the efficiency of RXR binding at PPARγ sites is augmented in the presence of its heterodimerization partner. Though this mechanism has been suggested in studies involving individual binding sites, our genome-wide analysis suggests that the PPARγ/RXR heterodimer is a general requirement for optimal DNA binding throughout the genome.

In addition, our data suggest asymmetric requirements for PPARγ and RXR in this heterodimerization process. RXR appeared to have an almost 4 fold higher ratio of high quality to lower confidence occupancy sites than PPARγ. Moreover, de novo motif analysis showed RXR binding sites gave a robust direct repeat that resembled the canonical PPRE whereas the PPARγ motif appeared more degenerate.

Our global results have revealed other general characteristics of PPARγ/RXR binding as it relates to downstream function. First, that proximity of a binding site to the TSS along with the level of occupancy of the heterodimer predicts for association with regulated genes, and in particular upregulated genes. We have observed this with other transcription factors such as ERα, Myc, and p53 in that proximity to the TSS (up to 50 kb) is statistically associated with upregulated genes. Downregulated genes do not have this position association with binding sites of their cognate transcription factors. When coupled with the observation that the evolutionary conservation of PPARγ/RXR binding sites is greater when adjacent to upregulated genes than downregulated genes suggest that the primordial function of PPARγ/RXR is the induction of genes involved in lipid and fatty acid metabolism.

Extending the functional importance of PPARγ/RXR transcriptional regulation to adipogenesis, our assessment of the ontologic classification of the genes in proximity to PPARγ/RXR heterosites shows significant enrichment for three major classes. The most enriched are genes involved in lipid, fatty acid, and steroid metabolism, and then genes involved in apoptosis. In a broader analysis of all PPARγ and RXR binding sites, lipid, fatty acid, and steroid metabolism cluster remains the most enriched, with other metabolic processes following. This suggests a transcriptional network with PPARγ/RXR at its core mediating an appropriate transcriptional response to changing ligand (i.e. fatty acids) concentration by fine tuning the levels of enzymes involved in metabolic processes associated with fatty acid and lipid metabolism. A comparison of the functional classification of downstream targets of different transcription regulators is also informative. The function of downstream targets of transcription factors such as ERα, Oct4-Nanog, p53 and RELA [21], [23], [28], [36] include a significant number of other transcription factors suggesting a “cascade” effect on transcription as a result of the induction of a series of other transcriptional regulators. By contrast, however, transcription factors are not overrepresented among direct PPARγ targets. While this is not ruling out any crucial function for TFs in the process of adipogenesis downstream of PPARγ as was shown for C/EBPa and Stat5a, the enrichment of enzymes involved in metabolic processes is very significant.

Only a limited number of genes are known to date to be direct targets of PPARγ, with the majority being involved in lipid metabolism [27]. In this study we extended this number to about 250 putative target genes (Spreadsheets S3 and S6). On the assumption that these direct target genes may have a central role in the adipogenic action of PPARγ/RXR activation, we examined the biological significance of a number of direct target genes identified in our study in the adipocyte differentiation process. We hypothesized that by suppressing these direct targets of PPARγ using siRNAs targeted against them, we would be able to inhibit adipogenesis, hence justifying the importance of these direct targets in the PPARγ adipogenic cascade. Indeed, of the 20 putative direct targets genes assessed using siRNA approaches, we found that 6 or 30% showed inhibition of adipocyte differentiation of 3T3-L1 cells when expression was attenuated using gene specific siRNAs. Using this approach, we have demonstrated that selective inhibition of PPARγ direct targets do in fact play a crucial role in the PPARγ adipogenic cascade, even in the presence of endogenous PPARγ. These putative targets include genes previously associated with obesity, while others have not been well characterized. *Pim3*, which we demonstrate here as being a direct target of PPARγ is a serine threonine kinase that is evolutionarily highly conserved. Mice lacking expression of *Pim1*, *Pim2*, and *Pim3* although viable and fertile show profound reduction in body size at birth and throughout postnatal life [37]. Although the contribution of *Pim3* in mammalian development needs further investigation, its role as an oncogene is well documented. Aberrant *Pim3* expression in cancer cells mediates its anti-apoptotic effects by phosphorylating Bad [38], [39]. This is potentially significant, since PPARγ is also highly expressed in many cancers [40] and therefore may play a role in inhibiting apoptosis via *Pim3* expression. Additionally we have identified novel PPARγ direct targets that have previously been demonstrated to promote lipid accumulation, regulate lipolysis and promote triglyceride accumulation. These include FSP27/Cidec, which has previously been reported to be regulated by C/EBP and other C/EBP-like proteins and functions in regulating adipocyte metabolism and enhancing triglyceride storage. FSP27 is expressed at high levels in brown adipose tissue (BAT), liver and white adipose tissue (WAT), and its expression is enhanced during adipogenesis in primary rodent and human preadipocytes. In addition, insulin exposure enhanced FSP27 transcript levels [41], [42]. Recent evidence suggests FSP27 regulates lipid accumulation and triacylglycerol storage by binding to lipid droplets and regulating their enlargement [42], [43]. Adipogenin (SMAF1, BC054059), another direct PPARγ target, stimulates adipocyte differentiation and development and is exclusively expressed in adipocytes isolated from primary adipose tissues [44]. Recent evidence suggests transmembrane proteins play a vital role in the development of adipose tissue and obesity. The expression of adiponutrin, a transmembrane protein, is regulated in human adipose tissue by the level of energy balance in the body [45]. Adipogenin may function in a similar manner by regulating levels of lipid stores during adipogenesis. We have shown that the downregulation of angiotensinogen (Agt) inhibits adipogenesis in 3T3-L1 cells. AGT is secreted by WAT, is induced during adipogenic conversion, and may contribute to the elevation of blood pressure and several cardiovascular risk factors in obese patients [46], [47]. Lipid accumulation and adipocyte differentiation is severely impaired when angiotensinogen levels are reduced. Though the role of angiotensinogen in the differentiation of adipoblasts has not been elucidated, angiotensinogen signaling has been linked to leptin/leptin receptor signaling, insulin receptor signaling and the metabolism of complex and glycerolipids, via serpine1 [48]. GPRK7 (Mnk2), which phosphorylates eukaryotic initiation factor 4E and which we show here as being a direct target, also inhibits adipogeneis. The role of G-protein coupled receptors in adipocyte biology is unclear. Of the numerous GPR expressed, GPR120 and 43 has been linked with a possible physiological role in adipose tissue development and adipocyte differentiation. The levels of both receptors are highly expressed in adipose tissues of mice fed on a high fat diet, and siRNA targeting these receptors inhibited adipocyte differentiation [44], [49]. In addition to the gene targets that were transcriptional upregulated by the PPARγ/RXR heterodimer, we also investigated whether KD of target genes that were transcriptionally downregulated by the heterodimer could enhance adipogenesis. For this, we targeted PDZRN3, which contained a heterosite within its promoter and was downregulated by PPARγ during the course of adipogenesis. PDZRN3 is a PDZ domain containing RING finger 3 (PDZRN3) that potentially has E3 ubiquitin ligase activity. Previous studies have shown that PDZRN3 is expressed in the heart, skeletal muscle and liver. Up-regulation of PDZRN3 during differentiation of myoblasts into myotubes, and injury induced muscle regeneration has been described [50].

Although PDZRN3 has a role in myoblasts differentiation, we observe a paradoxical finding during adipocyte differentiation. We postulated that KD of PDZRN3 could enhance adipogenesis. Our siRNA screen for PDZRN3 resulted in a decrease in adipogenesis. This observation suggests a complex role for PDZRN3 in adipocyte differentiation which will require further study. The remaining direct targets of PPARγ/RXR that were tested showed no changes in lipid accumulation when targeted by their respective siRNAs. These targets may not contribute directly to the accumulation of lipids during the adipogenic process, but may play a role in other metabolic processes or may play a redundant role during adipocyte lipid accumulation and differentiation. Of the interesting targets that showed no phenotypic changes include, Mgst1 (MAPEG, Membrane Associated Proteins in Eicosanoid and Glutathione metabolism family member) that may be involved in inflammation and cellular defense and ECH1 (enoyl Coenzyme A hydratase 1) which is involved in fatty acid beta-oxidation [51]. The rest of the targets that were screened have not previously been reported to play a role in metabolic processes related to obesity. Our study provides a comprehensive source of PPARγ direct targets to the scientific community studying adipogenesis to further investigate the importance of this cascade sequence.

Taken together, our genome-wide analysis of the comprehensive DNA interaction of PPARγ and its heterodimerization partner, RXR provides an enhanced understanding of global functions of this NHR pair. Such a “population” approach helps resolve the core actions by filtering the evolutionary noise inherent in short motif recognition systems. Our observations suggest several models for PPARγ/RXR function that can now be tested using the comprehensive binding database that we are providing.

## Materials and Methods

### Antibodies and Reagents

Rosiglitazone was obtained from Cayman Chemical (Ann Arbor, MI, USA). 3-Isobutyl-1-methylxanthine and Dexamethasone was obtained from Sigma (St. Louis, MO, USA).

Primary antibodies- ApoA-1 (C-18):sc-19029, Adipsin (L-21): sc-12403, Ob (H-146):sc-9014, A-FABP (C-15): sc-18661, PPARγ (H-100): sc-7196 and secondary antibodiesgoat anti-mouse IgG-HRP, goat anti-rabbit IgG-HRP and donkey anti-goat IgG-HRP were from Santa Cruz Biotechnology (Santa Cruz, CA, USA).

### Cell culture, induction of adipocyte differentiation and Oil Red O staining

3T3-L1 cell line was purchased from ATCC (Manassas, VA, USA). Culture of 3T3-L1 cells and induction of differentiation was performed as described in the Adipogenesis Assay Kit (Chemicon International, Inc, USA).Briefly, differentiation of 3T3-L1 fibroblasts into adipocytes was performed by treating confluent cells with 0.5 mM isobutylmethylxanthine (IBMX) and 0.25 µM dexamethasone for 48 hrs and then with insulin (5 µg/ml) and 1 µM Rosiglitazone for 48 hrs. Media was replaced every 2 days. Cells were cultured and harvested 6 days post rosiglitazone treatment.

### siRNA Transfection

*SMART*pool siRNA and negative control siRNA (siCONTROL non-targeting pooled) were purchased from Dharmacon, Inc (Lafayette, CO, USA). Cells were transfected using the LipofectAMINE RNAimax reagent (Invitrogen) according to the manufacturer's protocol. Final concentration of siRNA was 50 nM.

### Immunoblot Analysis

For detection of cellular proteins, cells were treated with SDS sample buffer (62.5 mM Tris-HCl, 2% SDS, 10% glycerol, 50 mM dithiothreitol, 0.01% (w/v) bromophenol blue), sonicated for 15 s, heated for 5 min at 95°C, and cooled on ice. Cell extracts were electrophoresed on a 12% SDS-PAGE gel and transferred onto nitrocellulose membrane. Blocking buffer (1× TBS, 0.1% Tween 20, and 5%w/v nonfat dry milk) was added to the membrane for 30 minutes at room temperature. Primary antibodies were diluted in primary antibody dilution buffer (1×TBS, 0.1% Tween 20, 5% w/v BSA) and membranes were incubated overnight at 4°C. Membranes were washed with wash buffer (1× TBS, 0.1% Tween 20) and incubated with the appropriate secondary IgG-HRP antibody in blocking buffer for 30 minutes at room temperature. Proteins were detected using SuperSignal West Pico Chemiluminescent Substrate (Pierce, Rockford, IL).

### Chromatin Immunoprecipitation

Day 3 adipocytes were treated with 1 µM Rosi 24 hours prior to ChIP procedures. ChIP was carried out as described previously [23] using the H-100 anti-PPARγ antibody, ΔN 197 anti-RXR antibody or Rabbit IgG antibody (Santa Cruz). Following ChIP, DNA was analyzed for PPARγ and RXR binding at known PPARγ binding sites by real-time q-PCR.

### Quantitative PCR

PCR quantification was performed on the ABI7500 Real-time PCR System (Applied Biosystems) with 20 µL reaction volume consisting of ChIP samples immunoprecipitated with IgG, RXR or PPARγ antibodies, 0.2 µM primer pairs, and 10 µL of 2× SYBR Green PCR Master Mix (Applied Biosystems). For each PCR run, the samples underwent 40 amplification cycles. Fluorescence was acquired at the conclusion of each cycle at 60 C during the amplification step.

### ChIP-PET Library Construction and Sequencing

ChIP was performed as previously described [23] using antibodies for RXR and PPARγ (Santa Cruz, CA, USA). The ChIP-PET analysis was performed as previously described [21] using the UCSC mm8 genome as the reference genome. The locations of the ChIP-enriched DNA present in the two libraries were visualized using our in-house genome browser (T2G browser) which was implemented in the context of the University of California, Santa Cruz (UCSC) genome browser. ChIP-PET fragments overlapping by at least 1 basepair were iteratively clustered. Binding regions of PPARγ and RXR were identified from the set of all ChIP-PET clusters using adaptive thresholding method similar to [25]. In brief: the adaptive threshold method is based on a Monte-Carlo simulation. Significance for a binding site was determined by computing the probability of a peak with a certain size being observed in the library occurring by random chance, given the total number of peaks in a defined interval. The cut-off is therefore not fixed to a certain moPET number, for the entire library, but changes depending on the background in any given region.

ChIP-PET clusters that did not meet the adaptive thresholding criteria but were at least of moPET2 and above are considered low confidence sites. Closest genes were identified by using coordinates for 5′ and 3′ position. Coordinates were extracted from UCSC Known gene database or RefSeq annotated database to match against genes present on the array.

### Library Saturation Analysis

To assess the sampling adequacy of our libraries, we carried sequencing saturation analyses on both the PPARγ and RXR ChIP-PET libraries. A total of 3,211,429 and 1,646,083 PET reads were generated for the PPARγ and RXR libraries respectively, which identified around 549,000 unique ChIP fragment locations for PPARγ library and around 348,000 unique ChIP fragment locations for RXR library. Using the chronological information available from the sequencing pipeline coupled with Monte Carlo simulations and similar to what was done in [21]. We fitted a Hill Function to each empirical sequencing saturation curve to estimate the asymptotic identifiable unique locations. The saturation level was computed by taking the ratio of identified unique locations over the total identifiable unique locations. A saturation level of ∼63% and ∼65% were reached for PPARγ and RXR libraries respectively. The saturation curves are shown in Figure S2.

### Expression analysis

Total RNA was extracted using RNAeasy Kit (Qiagen). Biotin-labeled cRNA was prepared using the Illumina TotalPrep RNA amplification kit (Ambion) following the manufacturer's instructions. cRNA was hybridized to Sentrix MouseRef-8 Expression BeadChips. BeadChips were scanned and probe intensities were measured. Probe intensities were normalized using Rank invariant normalization (BeadStudio, Illumina). For statistical analysis of expression changes between PPARγ siRNA and control siRNA treated cell we used PARTEK's Genomics Suite. We employed 3-way ANOVA to eliminate an obvious batch effect and used corrected expression values thereafter. Probes with q≤0.05 (Bonferroni corrected) were considered significantly changed.

Association of regulated genes with specific biological processes was assessed using PANTHER (http://www.pantherdb.org/). List of differentially regulated gene was uploaded and significance of enrichment was calculated by comparing the number of genes associated with a given process to the number of genes expected to be associated on the basis on a genome wide reference list (NCBI Mus musculus, 29917 genes). P-values were Bonferroni corrected for multiple testing.

### Constuction of plasmids and Luciferase Reporter Assays

PPARγ/RXR ChIP-PET binding sites were amplified from 3T3-L1 genomic DNA by PCR and cloned into the pGL4-TATA vector (a minimal TATA box upstream of pGL4- Basic) by homologous recombination using the In-Fusion CF Dry-Down PCR Cloning kit (Clontech, http://www.clontech.com). Putative PPREs were mutated using the QuickChange Site Directed Mutagenesis kit according to the manufacturer's instructions (Stratagene, http://www.stratagene.com). 3T3-L1 cells were cotransfected with the ChIP-PET constructs, HSV-TK renilla and PPARγ expression vector with Lipofectamine 2000 (Invitrogen). After the cells were treated with 1 uM Rosiglitazone or DMSO for 18–24 h, cell lysates were harvested and assessed for firefly and renilla luciferase activity using the Dual Luciferase Reporter Assay system (Promega, http://www.promega.com).

### Assessing Significance of Distance Distribution

Genomic proximity between distinct elements can be used as a good proxy for functional interplays between the elements. We were interested to ascertain: (i) whether the identified PPARγ and RXR binding sites are preferentially concentrated around Transcription Start Sites (which suggests direct gene regulatory role), and (ii) whether the reported cooperation of PPARγ and RXR is evident at the genome scale. For the first task, we compiled the distances of all sites to all TSS (based on UCSC knownGene database) within 100 kbp away and grouped them into 5 kbp bins. As a control, the same amount of sites was randomly distributed in the genome and their distances to all TSS were similarly compiled and binned. Plotted in Figure 3B is the ratio, for each distance bin, of the binding site density from the actual data to that of the randomly distributed control.

Similarly, for the second task, distances of all PPARγ sites to all RXR sites were computed and split into 200 bp bins. Figure 3C shows the ratio of the observed occurrences in each bin to the expected occurrences had the sites been distributed randomly and independently. In addition to that, we estimated the Monte-Carlo False Discovery Rate (FDR) of PPARγ and RXR binding sites co-occurrence within varying distances (Figure 3C).

### Motif enrichment

To determine the presence of PPARγ-RXR binding site motifs (DR1) in the PPARγ and RXR ChIP-PET genome-wide binding site data sets, we analyzed the cluster sequences using the Matinspector program, which is part of the Genomatix software suite (Genomatix, Munich). The binding site predictions are based on a position weight matrix algorithm and to compute the statistical significance, we generated a background sequence set of 10,000 randomly selected 500 bp sequences, roughly matching the 9002 binding sites identified in the PPARγ and RXR ChIP-PET experiments. The analyzed ChIP-PET cluster sequences were 500 bp long, and defined using the center of the PET cluster as the middle of the cluster and then expanding 250 bp on each side. The p-values were computed under the binomial distribution and were adjusted for multiple hypotheses testing using the conservative Bonferroni correction.

### Calculation of Percent binding sites associated with PhastConsElements

For this analysis, we considered 20 bp binding regions centered on the middle of ChIP-PET overlap. Binding region within 5 kb from TSS of genes present in Illumina beadChip for which the coordinates could be located were split into regulated and non regulated genes. Based on the expression profile of the nearest gene, the binding regions were grouped into those associated with PPARγ-regulated and non-regulated genes. A binding region was said to be associated with conserved element if it overlaps with the PhastConsElement, obtained from UCSC database.

### De- novo motif construction

The *de-novo* motif discovery was carried out using Weeder [29] on 100 bp sequences centered on the middle of heterosites peaks, PPARγ monosites, and RXR monosites. To overcome the 12 bp limitation of Weeder, *de-novo* motifs that contained a pattern matching to at least a half-site of the known PPRE consensus were extended to a reasonable length that would contain the full PPRE consensus and were refined in an EM-like optimization using random genomic sequences as background [28].

## Supporting Information

Figure S1 Gene expression dynamics and Biological Processes during adipogenesis. Expression changes during adipogenesis are hallmarked by PPARγ induced genes and are biologically meaningful. ANOVA with a 5% FDR cutoff was used to find genes differentially expressed between 3T3-L1 cells treated with PPARγ specific siRNA and control siRNA, respectively. A) Proportion of genes significantly up- or down-regulated, defined as having a fold difference >1.5, at each time point. B) Results of biological process analysis using PANTHER [http://www.pantherdb.org/]. A number of biological processes are significantly enriched among genes repressed (left panel) or induced (right panel) by PPARγ during adipogenesis. Statistical significance was computed by comparing the number of genes in each category to expected number derived from the total number of genes in each process using NCBI mus musculus Ref Seq as reference. P-values were Bonferroni corrected for multiple hypotheses testing. Genes were categorized as repressed or induced according to their average fold change throughout the time course.
(0.06 MB DOC)

Figure S2 Library saturation analysis. Results from library saturation analysis showed that (a) the PPARγ library was ∼63% saturated, while (b) the RXR library was ∼65% saturated. The x-axis shows the amount of sequence reads collected and the y-axis indicates the total unique genomic location obtained. The Hill Function was used as analytical curve to determine the asymptotic unique location attainable within the library.
(0.04 MB DOC)

Figure S3 PPARγ/RXR heterodimer is a general requirement for optimal DNA binding. High confidence targets were chosen for binding of PPARγ and RXR in undifferentiated (fibroblasts) and fully differentiated (adipocytes) 3T3-L1 cells. Binding was confirmed by ChIP Q-PCR. Values for fold enrichment of target genes over unspecific antibody control (rabbit IgG) represent the mean of three biological experiments, error bars indicate standard deviations.
(0.04 MB DOC)

Figure S4 Significant motifs found in heterosites and monosites. Sequence logos depicting the significant motifs found in the heterosites, RXR monosites, and PPARγ monosites. All the extended motifs encode the half-site of PPARγ binding elements. Only the motifs originated from heterosites and RXR monosites, however, seem to contain the full PPARγ binding elements.
(0.05 MB DOC)

Figure S5 Binding sites are associated with phastCons Elements. Binding sites (PPARγ/RXR, PPARγPET2/RXR, PPARγ, RXR, PPARγpet1, RXRpet1) within 5 kb of a TSS were analyzed for association with PhastCons Elments (UCSC genome browser; http://genome.ucsc.edu/). PPARγ/RXR heterosites showed a stronger association with phastChonsElements close to regulated genes then non regulated genes. Other binding categories showed higher association with conserved elements than PPARγpet1 and RXRpet1 as background.
(0.03 MB DOC)

Figure S6 Luciferase assays showing PPARγ dependent activation of luc reporter in 3T3-L1 fibroblasts. Putative PPARγ/RXR binding regions in proximity to Plin, PCX, Mgst1,Gpd1, Cops7b, Sncg and Pim3 were cloned into TATA box containing pGL3 reporter construct. Reporter constructs were co-transfected with PPARγ expression vector or empty vector as control. Putative PPRE in binding region adjacent to Pim3 and SNCG were mutated to confirm functionality (primers used in PCR amplification and cloning are listed in Table S5, together with their genomic locations). Samples were treated with 1 uM Rosiglitazone or DMSO for 24 h prior to quantization. Luciferase activity was measured and normalized to Renilla and cells transfected with minimal TATA luciferase construct. Mutation of putative PPARγ/RXR binding motif within the binding region abrogates PPARγ dependent activation of the luciferase reporter construct (mutations sites are highlighted in red). Shown is the average of two biological replicates each run in triplicates; with the standard deviations indicated as error bars. Asterix denotes p-values<0.05 (Student T-test, two tailed distribution).
(0.12 MB DOC)

Figure S7 Enrichment for PPARγ and RXR correlates at almost all tested sites. Correlation betweenPPARγ and RXR qChIP enrichment on randomly chosen PPARγ and RXR PET4+ monosites, respectively, as well as on PPARγ/RXR heterosites. Plot shows a high degree of correlation between the enrichment for PPARγ and RXR as measured by ChIPqPCR at most sites
(0.03 MB DOC)

Figure S8 Distance between middle of PPARγ and RXR moPET clusters and heterosites correlates with Chip enrichment and PET count. Enrichment in qPCR as well as moPET count for both PPARγ and RXR for heterosites is negatively correlated with the distance between the two binding partners. This is a qualitative statement to illustrate the characteristics of the heterosites. It suggests however that the observed distance between the peaks of the two binding sites decreases as the enrichement (occupancy) gets better. Essentially, the better the resolution at the individual binding site the better the overlap. Ideally the peak of two binding sites should overlap.
(0.16 MB DOC)

Figure S9 Binding sites close to regulated genes show a higher degree of motif occurrence. We scanned PPARγ, RXR binding regions and PPARγ/RXR binding regions (1 kb with the middle of the moPET region in the centre) for the occurrence of a published PPARγ consensus motif (AGGTCAAAAGGTCA) while allowing for up to 3 mismatches. Ideally the motif is to be expected to be located at the center of the cluster overlap (i.e. at the middle of the peak). However, depending on the cluster size the resolution might decrease. In addition, not all sites contain a motif or the motif is degenerated. When plotting the motif occurrence over the 1 kb window as density function, we found that RXR binding sites as well as PPARγ/RXR binding regions showed a marked peak in motif density around the centre of the binding regions. PPARγ binding regions showed a motif enrichment around the centre of the binding region as well, however this enrichment appeared to be somewhat weaker. When considering binding regions closer to genes all binding regions showed further increase in motif occurrence with the maximum for binding sites within 5 kb of regulated genes.
(0.16 MB DOC)

Figure S10 RXR binding sites are used to improve grading of PPARγ binding sites and reduce false negative rate in PPARγ data. Schematic illustration of our approach to consider PPARγPET2+/RXR heterosites as high confidence sites: RXR is used as quality binding site to ‘fish’ for a PPARγ site within close proximity that was not detected using the adaptive threshold method. This helps to utilize the PPARγ PET2+ cluster and hence reduces the false negative rate. We used these sites to identify direct targets.
(0.05 MB DOC)

Figure S11 Genomic distribution of PPARγ moPET1 and RXR moPET1 fragments gives an approximate background distribution. Fragments were pooled for a composite distribution chart in Fig 3A.
(0.08 MB DOC)

Table S1 Biological process analysis of direct PPARγ target genes. Results of biological process analysis for direct PPARγ target genes using PANTHER [http://www.pantherdb.org/]. Genes were considered putative direct targets if at least one binding site from any category (heterosites, PPARγ monosites, RXR monosites) was found within 5 kb of their TSS. TSS coordinates were extracted from UCSC database KnownGenes. Statistical significance was computed by comparing the number of genes in each category to expected number derived from the total number of genes in each process using NCBI mus musculus Ref Seq as reference. P-values were Bonferroni corrected for multiple hypotheses testing. Genes were categorized as repressed or induced according to their average fold change throughout the time course.
(0.04 MB DOC)

Table S2 Percent of regulated genes having binding sites. Number of binding sites in proximity to genes is shown for different classes of binding sites. Table lists total no. of binding sites in proximity to a gene as opposed to no. of genes targeted by PPARγ. Gene coordinates were extracted from UCSC RefGene Database.
(0.03 MB DOC)

Table S3 Calculation of PPARγ/RXR binding motif occurrence. PPARγ/RXR binding motif occurrence was evaluated in different sets of binding sites. All PPARγ, RXR and conjoint PPARγ2/RXR binding regions (500 bp) as well as binding regions in proximity to regulated genes were screened for occurrence of a described PPARγ binding motif (PERO) using Genomatix. The no. of occurrences is listed. The binomial p-value of motif enrichment in the particular categories was calculated using 10000 randomly chosen sites.
(0.03 MB DOC)

Table S4 Comparison of ChIP-qPCR enrichments for the different groups of binding sites. (A) No statistically significant difference was observed between ChIP-qPCR enrichment of PPARγ andRXR across all groups of binding sites. (B) ChIP-qPCR enrichment (on both PPARγ and RXR antibodies) among the heterosites was significantly higher than those of monosites, although weak heterosites was of less significance.
(0.03 MB DOC)

Table S5 Primers used to amplify genomic regions for luciferase constructs. Primers were selected to amplify 500 bp around the binding regions of PPARg and RXR.
(0.05 MB DOC)

Table S6 Pathways implicated by PPARγ PET2+/RXR heterosites. Significant association of Pathways (PANTHER) with genes regulated during adipogenesis which are in proximity (5 kb) to PPARγ PET2+/RXR heterosites.
(0.15 MB DOC)

Table S7 Pathways implicated by PPARγ sites. Significant association of Pathways (PANTHER) with genes regulated during adipogenesis which are in proximity (5 kb) to PPARγ sites.
(0.17 MB DOC)

Table S8 Pathways implicated by RXR sites. Significant association of Pathways (PANTHER) with genes regulated during adipogenesis which are in proximity (5 kb) to RXR sites
(0.28 MB DOC)

Spreadsheet S1 Day −2 to 6 differentially expressed genes. Genes that are differentialy expressed during adipocyte differentiation between cells treated with siRNA non-targeting versus siRNA PPARg
(0.15 MB XLS)

Spreadsheet S2 Day 4 to 6 differentially expressed genes. Genes that are differentialy expressed during adipocyte differentiation on day 4 to 6 between cells treated with siRNA non-targeting versus siRNA PPARg
(0.07 MB XLS)

Spreadsheet S3 PPARg clusters (2953). Location of PPARg binding clusters
(0.33 MB XLS)

Spreadsheet S4 RXR clusters (5142). Location of RXR binding clusters
(0.57 MB XLS)

Spreadsheet S5 PPAR_RXR_200 bp with annotation (907). PPARg binding clusters that overlap with RXR binding clusters that are within 200 bp of each other
(0.19 MB XLS)

Spreadsheet S6 Binding sites that are close to genes combined with annotation. Binding clusters for PPARg or RXR that are close to annotated genes
(0.07 MB XLS)
