# Supplementary figures and images for: De-Novo Identification of PPARγ/RXR Binding Sites and Direct Targets during Adipogenesis
Source: PLoS One. 2009 Mar 20;4(3):e4907. doi: 10.1371/journal.pone.0004907 (PMC2654672; doi:10.1371/journal.pone.0004907)

**Figure S1.** Gene expression dynamics and Biological Processes during adipogenesis.


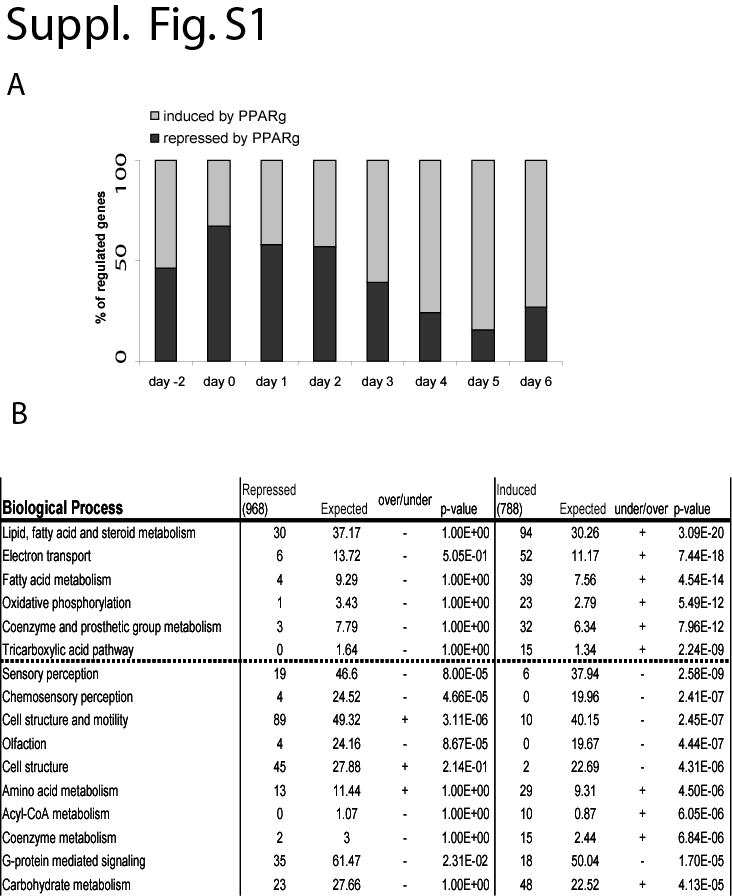

Supplement: Figure S1 — Gene expression dynamics and Biological Processes during adipogenesis. Expression changes during adipogenesis are hallmarked by PPARγ induced genes and are biologically meaningful. ANOVA with a 5% FDR cutoff was used to find genes differentially expressed between 3T3-L1 cells treated with PPARγ specific siRNA and control siRNA, respectively. A) Proportion of genes significantly up- or down-regulated, defined as having a fold difference >1.5, at each time point. B) Results of biological process analysis using PANTHER [http://www.pantherdb.org/]. A number of biological processes are significantly enriched among genes repressed (left panel) or induced (right panel) by PPARγ during adipogenesis. Statistical significance was computed by comparing the number of genes in each category to expected number derived from the total number of genes in each process using NCBI mus musculus Ref Seq as reference. P-values were Bonferroni corrected for multiple hypotheses testing. Genes were categorized as repressed or induced according to their average fold change throughout the time course. (0.06 MB DOC) [file pone.0004907.s001.doc]

**Figure S2**. Library saturation analysis.


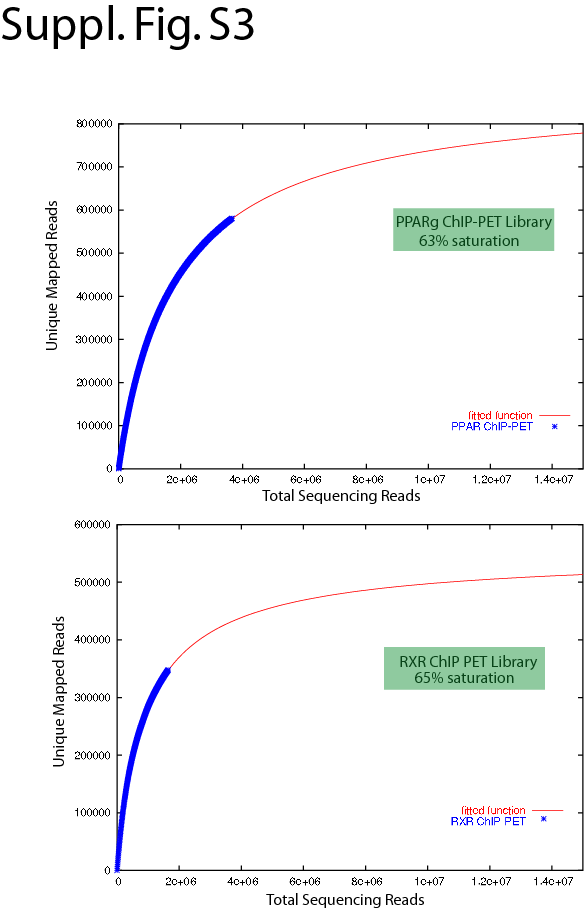

Supplement: Figure S2 — Library saturation analysis. Results from library saturation analysis showed that (a) the PPARγ library was ∼63% saturated, while (b) the RXR library was ∼65% saturated. The x-axis shows the amount of sequence reads collected and the y-axis indicates the total unique genomic location obtained. The Hill Function was used as analytical curve to determine the asymptotic unique location attainable within the library. (0.04 MB DOC) [file pone.0004907.s002.doc]

**Figure S3.** PPARγ/RXR heterodimer is a general requirement for optimal DNA binding.


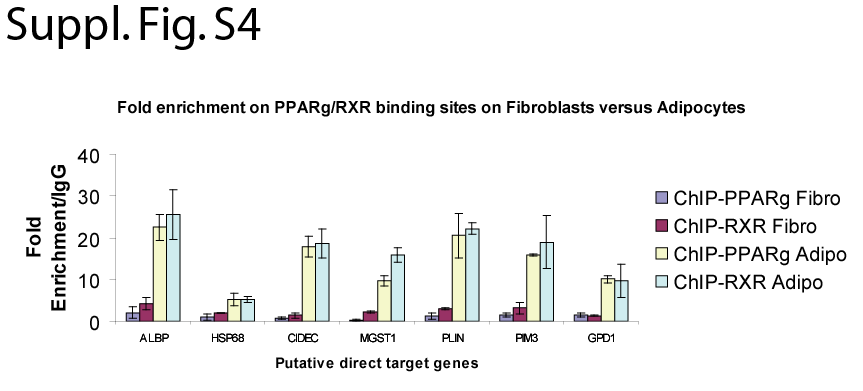

Supplement: Figure S3 — PPARγ/RXR heterodimer is a general requirement for optimal DNA binding. High confidence targets were chosen for binding of PPARγ and RXR in undifferentiated (fibroblasts) and fully differentiated (adipocytes) 3T3-L1 cells. Binding was confirmed by ChIP Q-PCR. Values for fold enrichment of target genes over unspecific antibody control (rabbit IgG) represent the mean of three biological experiments, error bars indicate standard deviations. (0.04 MB DOC) [file pone.0004907.s003.doc]

**Figure S4.** Significant motifs found in heterosites and monosites.


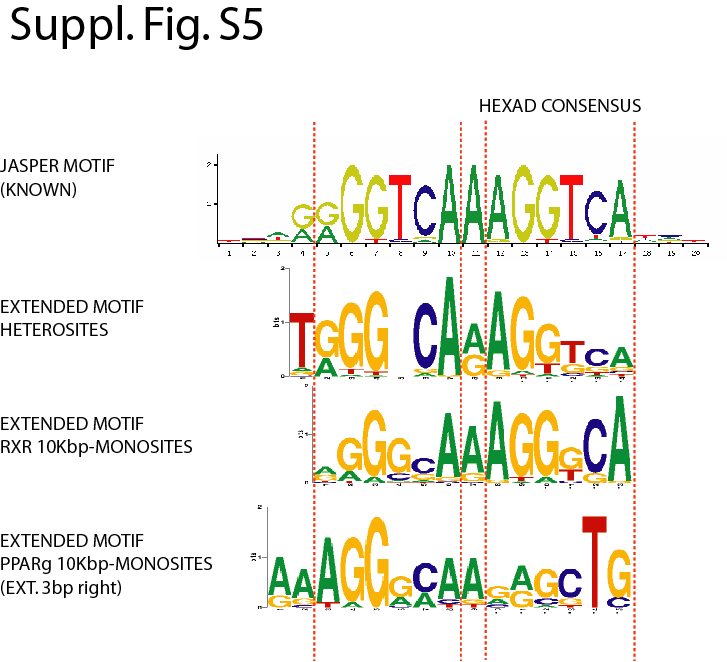

Supplement: Figure S4 — Significant motifs found in heterosites and monosites. Sequence logos depicting the significant motifs found in the heterosites, RXR monosites, and PPARγ monosites. All the extended motifs encode the half-site of PPARγ binding elements. Only the motifs originated from heterosites and RXR monosites, however, seem to contain the full PPARγ binding elements. (0.05 MB DOC) [file pone.0004907.s004.doc]

**Figure S5.** Binding sites are associated with phastCons Elements.
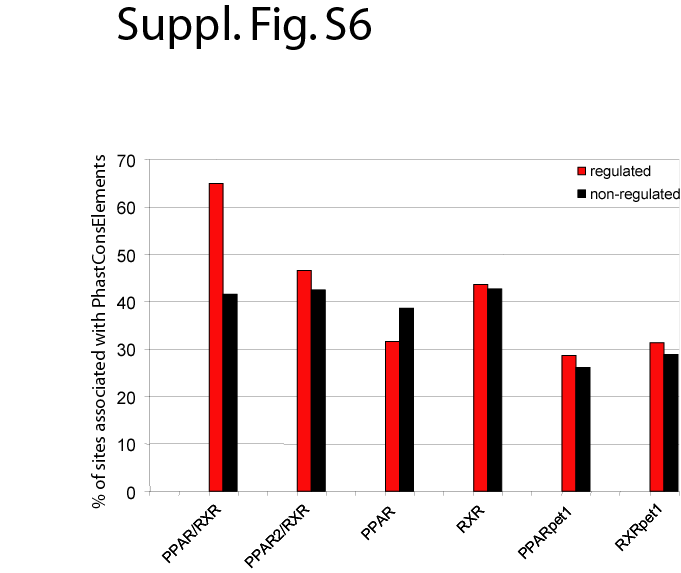

Supplement: Figure S5 — Binding sites are associated with phastCons Elements. Binding sites (PPARγ/RXR, PPARγPET2/RXR, PPARγ, RXR, PPARγpet1, RXRpet1) within 5 kb of a TSS were analyzed for association with PhastCons Elments (UCSC genome browser; http://genome.ucsc.edu/). PPARγ/RXR heterosites showed a stronger association with phastChonsElements close to regulated genes then non regulated genes. Other binding categories showed higher association with conserved elements than PPARγpet1 and RXRpet1 as background. (0.03 MB DOC) [file pone.0004907.s005.doc]

**Figure S11.** Genomic distribution of PPARγ moPET1 and RXR moPET1


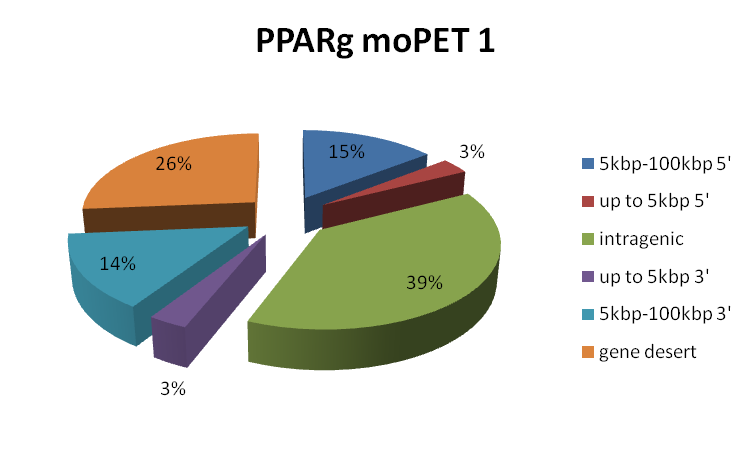

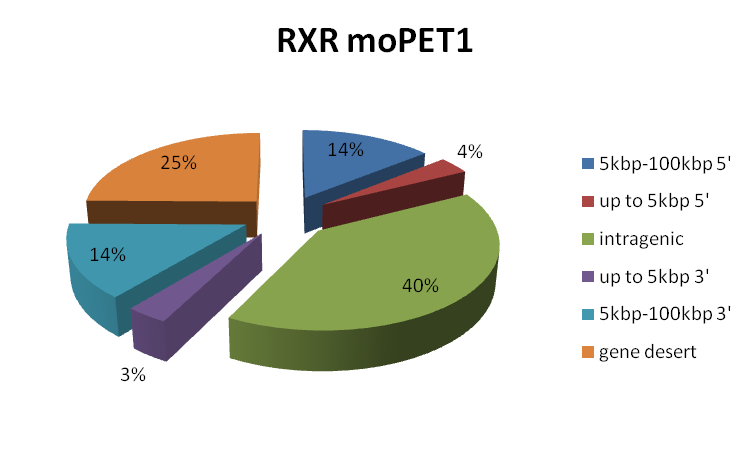

Supplement: Figure S11 — Genomic distribution of PPARγ moPET1 and RXR moPET1 fragments gives an approximate background distribution. Fragments were pooled for a composite distribution chart in Fig 3A. (0.08 MB DOC) [file pone.0004907.s011.doc]
