# Supplementary material for: De-Novo Identification of PPARγ/RXR Binding Sites and Direct Targets during Adipogenesis
Source: PLoS One. 2009 Mar 20;4(3):e4907. doi: 10.1371/journal.pone.0004907 (PMC2654672; doi:10.1371/journal.pone.0004907)
Supplement: Figure S6 — Luciferase assays showing PPARγ dependent activation of luc reporter in 3T3-L1 fibroblasts. Putative PPARγ/RXR binding regions in proximity to Plin, PCX, Mgst1,Gpd1, Cops7b, Sncg and Pim3 were cloned into TATA box containing pGL3 reporter construct. Reporter constructs were co-transfected with PPARγ expression vector or empty vector as control. Putative PPRE in binding region adjacent to Pim3 and SNCG were mutated to confirm functionality (primers used in PCR amplification and cloning are listed in Table S5, together with their genomic locations). Samples were treated with 1 uM Rosiglitazone or DMSO for 24 h prior to quantization. Luciferase activity was measured and normalized to Renilla and cells transfected with minimal TATA luciferase construct. Mutation of putative PPARγ/RXR binding motif within the binding region abrogates PPARγ dependent activation of the luciferase reporter construct (mutations sites are highlighted in red). Shown is the average of two biological replicates each run in triplicates; with the standard deviations indicated as error bars. Asterix denotes p-values<0.05 (Student T-test, two tailed distribution). (0.12 MB DOC) [file pone.0004907.s006.doc]

**Figure S6.** Luciferase Assay on PPARg/RXR binding sites

PPARg EXPRESS VECTOR

EMPTY EXPRESS VECTOR

MUT LUC VECTOR

WT LUC VECTOR

MIN LUC VECTOR

ROS

DMSO

**Min TATA Luc**

**vector**

**Plin Luc**

**vector**

**PCX Luc**

**vector**

**Mgst1 Luc**

**vector**

**Gpd1 Luc**

**vector**

**Cops7b Luc**

**vector**

**Sncg Luc**

**vector**

**Pim3 Luc**

**vector**

**Fold activity over renila (Log Scale)**

*

*

*

*

**Sncg WT** AGGAAACCAAAGCAAAGGTTAGGAATTTTCCCAGGACTT

**Sncg MUT** AGGAAACCAAAGCAAA**C**GTT**C**GGAATTTTCCCAGGACTT

**Pim3 WT**  GGCAGTCACAGTGGCTGGGCCAGAGTTCATGCAGGCTGC

**Pim3 MUT** GGCAGTCACAGTGGCTG**T**GCCAG**T**GTTCATGCAGGCTGC
