# Supplementary material for: De-Novo Identification of PPARγ/RXR Binding Sites and Direct Targets during Adipogenesis
Source: PLoS One. 2009 Mar 20;4(3):e4907. doi: 10.1371/journal.pone.0004907 (PMC2654672; doi:10.1371/journal.pone.0004907)
Supplement: Figure S7 — Enrichment for PPARγ and RXR correlates at almost all tested sites. Correlation betweenPPARγ and RXR qChIP enrichment on randomly chosen PPARγ and RXR PET4+ monosites, respectively, as well as on PPARγ/RXR heterosites. Plot shows a high degree of correlation between the enrichment for PPARγ and RXR as measured by ChIPqPCR at most sites (0.03 MB DOC) [file pone.0004907.s007.doc]

**Figure S7.** Enrichment for PPARγ and RXR correlates at almost all tested sites.
