# Supplementary material for: De-Novo Identification of PPARγ/RXR Binding Sites and Direct Targets during Adipogenesis
Source: PLoS One. 2009 Mar 20;4(3):e4907. doi: 10.1371/journal.pone.0004907 (PMC2654672; doi:10.1371/journal.pone.0004907)
Supplement: Figure S8 — Distance between middle of PPARγ and RXR moPET clusters and heterosites correlates with Chip enrichment and PET count. Enrichment in qPCR as well as moPET count for both PPARγ and RXR for heterosites is negatively correlated with the distance between the two binding partners. This is a qualitative statement to illustrate the characteristics of the heterosites. It suggests however that the observed distance between the peaks of the two binding sites decreases as the enrichement (occupancy) gets better. Essentially, the better the resolution at the individual binding site the better the overlap. Ideally the peak of two binding sites should overlap. (0.16 MB DOC) [file pone.0004907.s008.doc]

**Figure S8.** Distance between middle of PPARγ and RXR moPET clusters and heterosites correlates with ChIP enrichment and PET count.
