# Supplementary material for: De-Novo Identification of PPARγ/RXR Binding Sites and Direct Targets during Adipogenesis
Source: PLoS One. 2009 Mar 20;4(3):e4907. doi: 10.1371/journal.pone.0004907 (PMC2654672; doi:10.1371/journal.pone.0004907)
Supplement: Figure S9 — Binding sites close to regulated genes show a higher degree of motif occurrence. We scanned PPARγ, RXR binding regions and PPARγ/RXR binding regions (1 kb with the middle of the moPET region in the centre) for the occurrence of a published PPARγ consensus motif (AGGTCAAAAGGTCA) while allowing for up to 3 mismatches. Ideally the motif is to be expected to be located at the center of the cluster overlap (i.e. at the middle of the peak). However, depending on the cluster size the resolution might decrease. In addition, not all sites contain a motif or the motif is degenerated. When plotting the motif occurrence over the 1 kb window as density function, we found that RXR binding sites as well as PPARγ/RXR binding regions showed a marked peak in motif density around the centre of the binding regions. PPARγ binding regions showed a motif enrichment around the centre of the binding region as well, however this enrichment appeared to be somewhat weaker. When considering binding regions closer to genes all binding regions showed further increase in motif occurrence with the maximum for binding sites within 5 kb of regulated genes. (0.16 MB DOC) [file pone.0004907.s009.doc]

**Figure S9.** Binding sites close to regulated genes show a higher degree of motif occurrence.


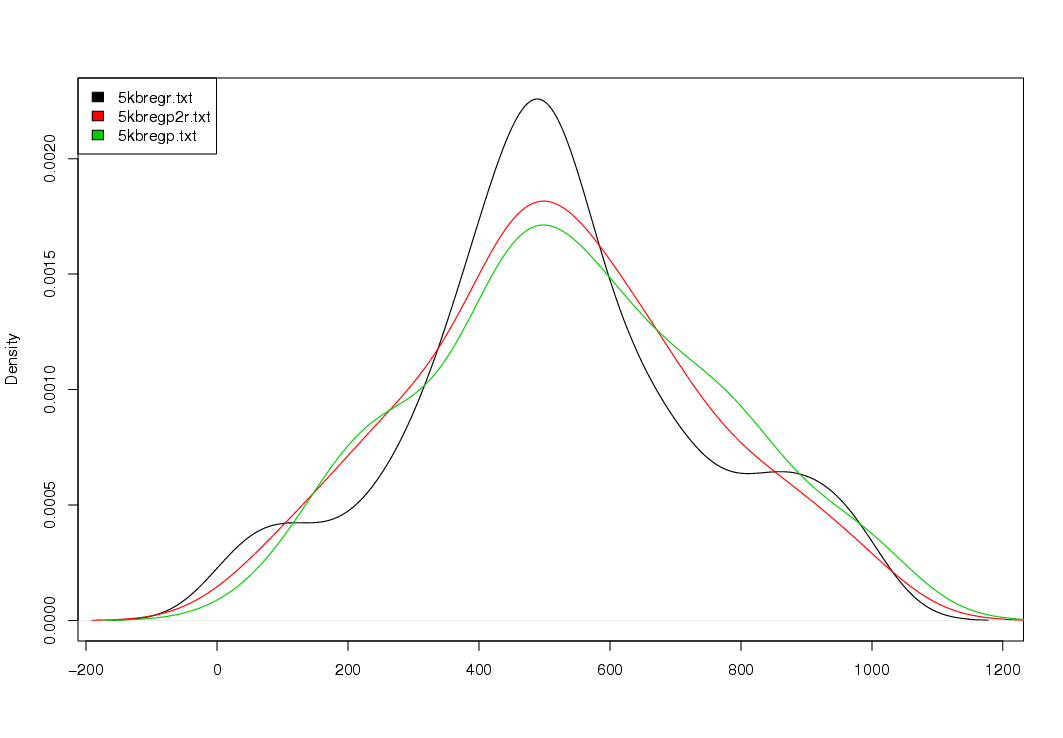

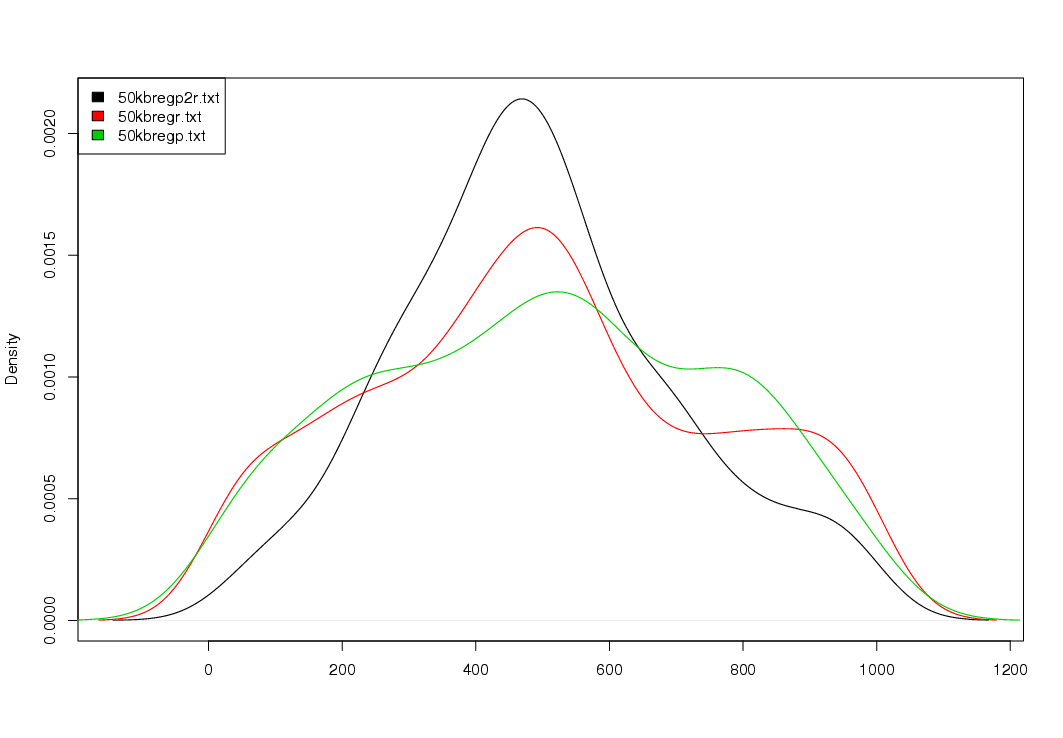

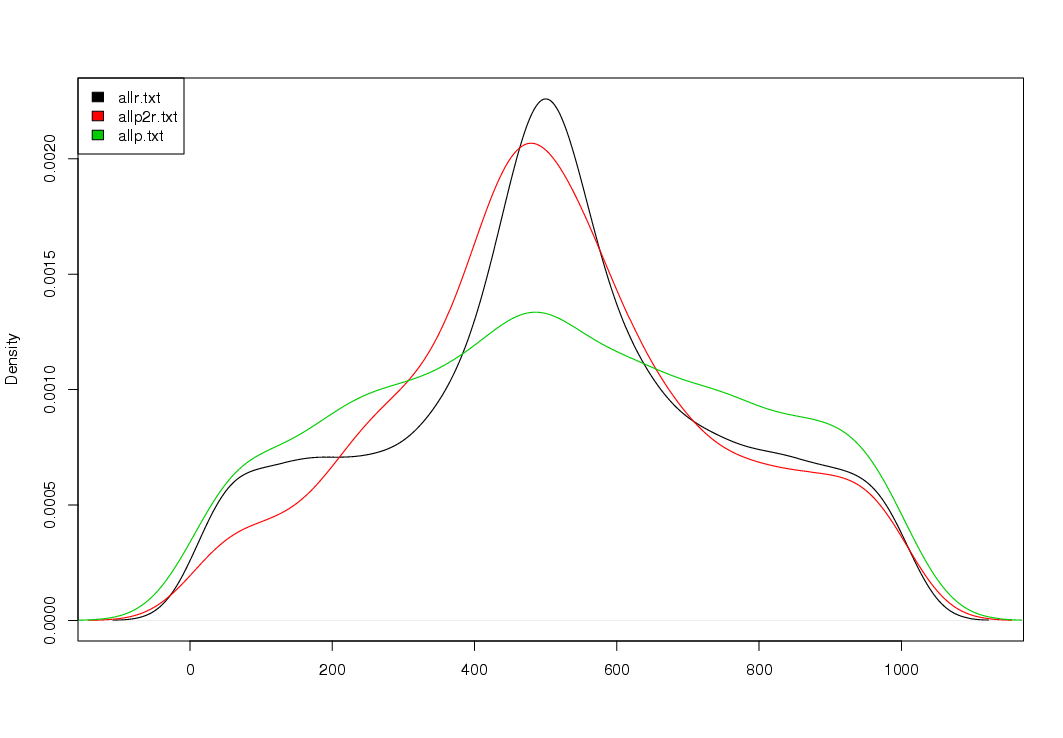

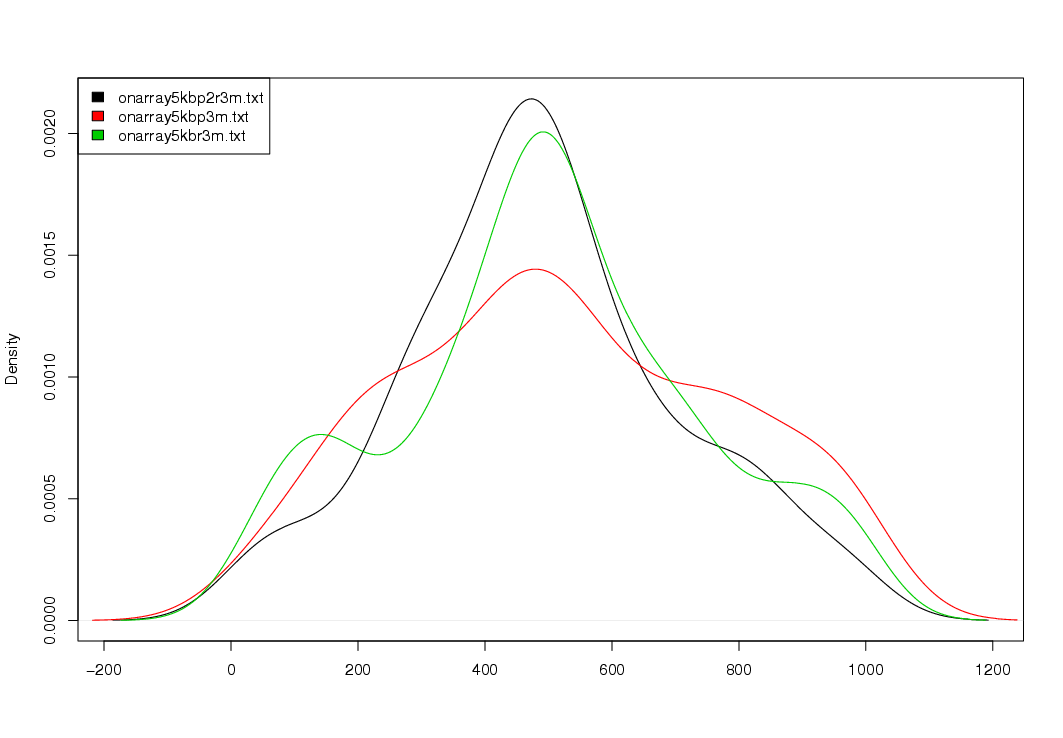


**All sites**

**Within 5kb of genes on**

**The array**

**Within 50 kb of**

**Regulated genes**

3 mismatches

Allowed to

AGGTCAAAGGTCA

**Consensus motif occurrence in different binding site populations**
