# Supplementary material for: De-Novo Identification of PPARγ/RXR Binding Sites and Direct Targets during Adipogenesis
Source: PLoS One. 2009 Mar 20;4(3):e4907. doi: 10.1371/journal.pone.0004907 (PMC2654672; doi:10.1371/journal.pone.0004907)
Supplement: Figure S10 — RXR binding sites are used to improve grading of PPARγ binding sites and reduce false negative rate in PPARγ data. Schematic illustration of our approach to consider PPARγPET2+/RXR heterosites as high confidence sites: RXR is used as quality binding site to ‘fish’ for a PPARγ site within close proximity that was not detected using the adaptive threshold method. This helps to utilize the PPARγ PET2+ cluster and hence reduces the false negative rate. We used these sites to identify direct targets. (0.05 MB DOC) [file pone.0004907.s010.doc]

**Figure S10.** RXR binding sites are used to improve grading of PPARγ binding sites and reduce false negative rate in PPARγ data.

**
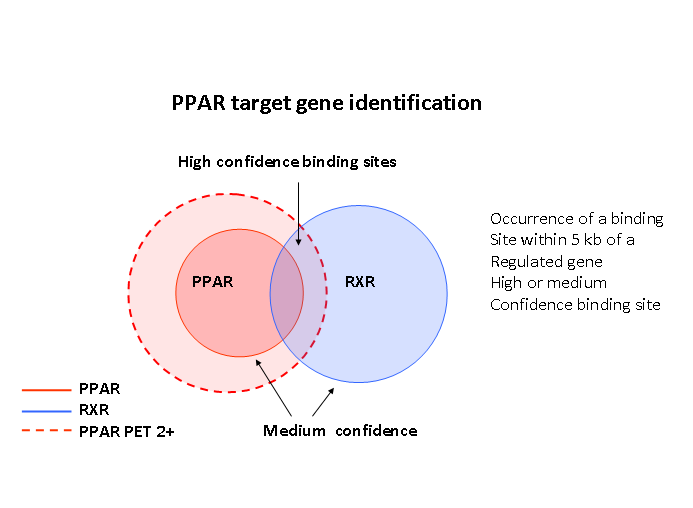
**
