# Supplementary material for: De-Novo Identification of PPARγ/RXR Binding Sites and Direct Targets during Adipogenesis
Source: PLoS One. 2009 Mar 20;4(3):e4907. doi: 10.1371/journal.pone.0004907 (PMC2654672; doi:10.1371/journal.pone.0004907)
Supplement: Table S1 — Biological process analysis of direct PPARγ target genes. Results of biological process analysis for direct PPARγ target genes using PANTHER [http://www.pantherdb.org/]. Genes were considered putative direct targets if at least one binding site from any category (heterosites, PPARγ monosites, RXR monosites) was found within 5 kb of their TSS. TSS coordinates were extracted from UCSC database KnownGenes. Statistical significance was computed by comparing the number of genes in each category to expected number derived from the total number of genes in each process using NCBI mus musculus Ref Seq as reference. P-values were Bonferroni corrected for multiple hypotheses testing. Genes were categorized as repressed or induced according to their average fold change throughout the time course. (0.04 MB DOC) [file pone.0004907.s012.doc]

**Table S1.** Biological process analysis of direct PPARγ target genes.

| **Biological Process** | **REF. List**  **(29917)** | **Targets Combined**  **(1551)** | **Expected** | **Over/Under Represented** | **P-value** |
| --- | --- | --- | --- | --- | --- |
| Lipid, fatty acid and steroid metabolism | 879 | 90 | 45.57 | + | 6.61E-08 |
| Nucleoside, nucleotide and nucleic acid metabolism | 3851 | 273 | 199.65 | + | 1.95E-06 |
| Cell proliferation and differentiation | 1004 | 88 | 52.05 | + | 7.02E-05 |
| Protein metabolism and modification | 3819 | 254 | 197.99 | + | 6.91E-04 |
| Carbohydrate metabolism | 608 | 57 | 31.52 | + | 7.11E-04 |
| Protein modification | 1300 | 104 | 67.4 | + | 1.95E-03 |
| Cell motility | 356 | 39 | 18.46 | + | 2.62E-03 |
| Apoptosis | 544 | 50 | 28.2 | + | 3.51E-03 |
| Stress response | 222 | 28 | 11.51 | + | 3.67E-03 |
| Phospholipid metabolism | 148 | 21 | 7.67 | + | 7.28E-03 |
| Fatty acid beta-oxidation | 24 | 8 | 1.24 | + | 9.29E-03 |
| Fatty acid metabolism | 224 | 27 | 11.61 | + | 1.07E-02 |
| Cell cycle | 1005 | 78 | 52.1 | + | 1.19E-02 |
| Intracellular protein traffic | 1044 | 80 | 54.12 | + | 1.46E-02 |
| rRNA metabolism | 90 | 15 | 4.67 | + | 1.53E-02 |
