# Supplementary material for: De-Novo Identification of PPARγ/RXR Binding Sites and Direct Targets during Adipogenesis
Source: PLoS One. 2009 Mar 20;4(3):e4907. doi: 10.1371/journal.pone.0004907 (PMC2654672; doi:10.1371/journal.pone.0004907)
Supplement: Table S2 — Percent of regulated genes having binding sites. Number of binding sites in proximity to genes is shown for different classes of binding sites. Table lists total no. of binding sites in proximity to a gene as opposed to no. of genes targeted by PPARγ. Gene coordinates were extracted from UCSC RefGene Database. (0.03 MB DOC) [file pone.0004907.s013.doc]

**Table S2.** Percent of regulated genes having binding sites.

**Sites within 5kb of gene Sites within 5kb of**

**on array regulated gene**

| **Sites** | **Total No. in this Category** | **Number** | **% Induced of total No.** | **Number** | **% Induced of total No.** |
| --- | --- | --- | --- | --- | --- |
| **PPARγ/RXR** | 272 | 53 | 19.5 | 26 | 9.6 |
| **PPARγPET2/RXR** | 907 | 188 | 20.7 | 75 | 8.3 |
| **PPAR** | 2953 | 350 | 11.9 | 79 | 2.7 |
| **RXR** | 5142 | 1027 | 20 | 256 | 5.0 |
