# Supplementary material for: De-Novo Identification of PPARγ/RXR Binding Sites and Direct Targets during Adipogenesis
Source: PLoS One. 2009 Mar 20;4(3):e4907. doi: 10.1371/journal.pone.0004907 (PMC2654672; doi:10.1371/journal.pone.0004907)
Supplement: Table S3 — Calculation of PPARγ/RXR binding motif occurrence. PPARγ/RXR binding motif occurrence was evaluated in different sets of binding sites. All PPARγ, RXR and conjoint PPARγ2/RXR binding regions (500 bp) as well as binding regions in proximity to regulated genes were screened for occurrence of a described PPARγ binding motif (PERO) using Genomatix. The no. of occurrences is listed. The binomial p-value of motif enrichment in the particular categories was calculated using 10000 randomly chosen sites. (0.03 MB DOC) [file pone.0004907.s014.doc]

**Table S3.** Calculation of PPARγ/RXR binding motif occurrence.

| **Cluster Type** | **Number of Clusters** | **Number of PPARγ/RXR motifs** | **Binomial P-value** |
| --- | --- | --- | --- |
| **PPARγ only** | 2953 | 1427(48.3%) | 2.68E-08 |
| **RXR only** | 5142 | 3258(63.4%) | 3.05E-183 |
| **PPARγ-RXR** | 907 | 685(75.5%) | 2.24E-19 |
| **<5kb of PPARγ down-regulated gene** | 81 | 51(63%) | 2.92E-04 |
| **<5kb of PPARγ up-regulated gene** | 226 | 158(69.9%) | 2.05E-16 |
