# Supplementary material for: De-Novo Identification of PPARγ/RXR Binding Sites and Direct Targets during Adipogenesis
Source: PLoS One. 2009 Mar 20;4(3):e4907. doi: 10.1371/journal.pone.0004907 (PMC2654672; doi:10.1371/journal.pone.0004907)
Supplement: Table S4 — Comparison of ChIP-qPCR enrichments for the different groups of binding sites. (A) No statistically significant difference was observed between ChIP-qPCR enrichment of PPARγ andRXR across all groups of binding sites. (B) ChIP-qPCR enrichment (on both PPARγ and RXR antibodies) among the heterosites was significantly higher than those of monosites, although weak heterosites was of less significance. (0.03 MB DOC) [file pone.0004907.s015.doc]

**Table S4.** Comparison of ChIP-qPCR enrichments for the different groups of binding sites.

**(A)**

| **Category** | **Avg. PPARγ**  **ChIP-qPCR** | **Avg. RXR**  **ChIP-q-PCR** | **Wilcoxon’s two-tailed p-value** |
| --- | --- | --- | --- |
| **PPARγ monosites** | 4.4 | 4.1 | 0.4357 |
| **RXR monosites** | 3.5 | 5.4 | 0.0145 |
| **Strong Heterosites** | 14.4 | 16.1 | 0.2792 |
| **Weak Heterosites** | 8.3 | 9.9 | 0.2380 |
| **Overall** | 8.2 | 9.5 | 0.1071 |

**(B)**

|  | PPARγ ChIP-qPCR | RXR ChIP-qPCR |
| --- | --- | --- |
| **Strong Heterosites vs. PPARγ monosites** | 4.57E-10 | 3.63E-11 |
| **Weak heterosites vs. PPARγ monosites** | 3.47E-05 | 1.67E-06 |
| **Strong heterosites vs. RXR monosites** | 1.77E-11 | 1.22E-09 |
| **Weak heterosites vs. RXR monosites** | 5.00E-07 | 3.80E-05 |
