# Supplementary material for: De-Novo Identification of PPARγ/RXR Binding Sites and Direct Targets during Adipogenesis
Source: PLoS One. 2009 Mar 20;4(3):e4907. doi: 10.1371/journal.pone.0004907 (PMC2654672; doi:10.1371/journal.pone.0004907)
Supplement: Table S5 — Primers used to amplify genomic regions for luciferase constructs. Primers were selected to amplify 500 bp around the binding regions of PPARg and RXR. (0.05 MB DOC) [file pone.0004907.s016.doc]

**Table S5. Primers used to amplify genomic regions for luciferase constructs.**

| **Target Gene of Promoter** | **Forward Primer** | **Reverse Primer** | **Genomic Coordinates** |
| --- | --- | --- | --- |
| PLIN | ACCGATAGATCCTTGGTAGT | CCTGGGATTGAAACGAAAAC | chr7:79608006-79608706 |
| SNCG | AGGGATTCGGCCTCTGCA | TCATGGACCAGAGAATGTC | chr14:33207770-33208470 |
| PIM3 | TTAGGCCTAGATACTCAT | ATCCCAGAAGGTACACCT | chr15:88685615-88686315 |
| PCX | TACAGCGTATACGAAGTCTG | TGTAACTGTTGGCATGTGGTT | chr19:4511250-4511950 |
| MGST1 | CTACAAGTCTTTACACTGGT | CTTGGGATGAAAGACTCCGA | chr6:138105324-138106024 |
| GPD1 | CACCCCTCGTAATCAGCGC | AAACAGCTGGTTACAGTCT | chr15:99543762-99544462 |
| COPS7B | TTAGTTGTTAGCATACTC | GGAGCCTCCAGGCGTCCT | chr1:88418033-88418333 |
