# Supplementary material for: De-Novo Identification of PPARγ/RXR Binding Sites and Direct Targets during Adipogenesis
Source: PLoS One. 2009 Mar 20;4(3):e4907. doi: 10.1371/journal.pone.0004907 (PMC2654672; doi:10.1371/journal.pone.0004907)
Supplement: Table S6 — Pathways implicated by PPARγ PET2+/RXR heterosites. Significant association of Pathways (PANTHER) with genes regulated during adipogenesis which are in proximity (5 kb) to PPARγ PET2+/RXR heterosites. (0.15 MB DOC) [file pone.0004907.s017.doc]

**Table S6.** Pathways implicated by PPARγ PET2+/RXR heterosites.

Targets (within 5Kb) of PPAR PET2+/RXR heterosites

Biological Process

REFLIST

(29917)

targets

(237)

(expected)

(over/under)

(P-value)

Lipid, fatty acid and steroid metabolism

879

18

6.96

+

8.17E-03

Apoptosis

544

13

4.31

+

1.45E-02

Fatty acid metabolism

224

8

1.77

+

6.91E-02

Sulfur redox metabolism

24

3

0.19

+

1.43E-01

Nucleoside, nucleotide and nucleic acid metabolism

3851

44

30.51

+

2.45E-01

Neuronal activities

841

1

6.66

-

2.83E-01

Biological process unclassified

12491

81

98.95

-

3.17E-01

Sensory perception

1229

3

9.74

-

3.50E-01

Purine metabolism

83

4

0.66

+

6.60E-01

Protein metabolism and modification

3819

41

30.25

+

8.25E-01

Cell proliferation and differentiation

1004

14

7.95

+

9.42E-01
