# Supplementary material for: De-Novo Identification of PPARγ/RXR Binding Sites and Direct Targets during Adipogenesis
Source: PLoS One. 2009 Mar 20;4(3):e4907. doi: 10.1371/journal.pone.0004907 (PMC2654672; doi:10.1371/journal.pone.0004907)
Supplement: Table S7 — Pathways implicated by PPARγ sites. Significant association of Pathways (PANTHER) with genes regulated during adipogenesis which are in proximity (5 kb) to PPARγ sites. (0.17 MB DOC) [file pone.0004907.s018.doc]

**Table S7.** Pathways implicated by PPARγ sites.

Biological Process

REFLIST

(29917)

Targets

(411)

(expected)

(over/

Under)

(P-value)

Biological process unclassified

12491

113

171.6

-

4.16E-08

Cell proliferation and differentiation

1004

35

13.79

+

2.17E-05

Lipid, fatty acid and steroid metabolism

879

28

12.08

+

1.40E-03

Apoptosis

544

20

7.47

+

2.72E-03

Sensory perception

1229

4

16.88

-

5.07E-03

Protein metabolism and modification

3819

78

52.47

+

6.99E-03

Receptor protein tyrosine kinase signaling pathway

218

12

2.99

+

1.27E-02

Other metabolism

627

19

8.61

+

4.09E-02

Fatty acid metabolism

224

11

3.08

+

4.88E-02

Nucleoside, nucleotide and nucleic acid metabolism

3851

74

52.91

+

5.67E-02

Granulocyte-mediated immunity

71

6

0.98

+

7.38E-02

Proteolysis

1151

30

15.81

+

1.06E-01

G-protein mediated signaling

1772

10

24.34

-

1.33E-01

Angiogenesis

54

5

0.74

+

1.97E-01
