# Supplementary material for: De-Novo Identification of PPARγ/RXR Binding Sites and Direct Targets during Adipogenesis
Source: PLoS One. 2009 Mar 20;4(3):e4907. doi: 10.1371/journal.pone.0004907 (PMC2654672; doi:10.1371/journal.pone.0004907)
Supplement: Table S8 — Pathways implicated by RXR sites. Significant association of Pathways (PANTHER) with genes regulated during adipogenesis which are in proximity (5 kb) to RXR sites (0.28 MB DOC) [file pone.0004907.s019.doc]

**Table S8.** Pathways implicated by RXR sites.

Biological Process

REFLIST

(29917)

Targets

(1255)

(expected)

(over/under)

(P-value)

Biological process unclassified

12491

403

523.99

-

4.10E-11

Sensory perception

1229

12

51.56

-

6.29E-10

Chemosensory perception

592

1

24.83

-

4.88E-08

Olfaction

586

1

24.58

-

8.53E-08

G-protein mediated signaling

1772

30

74.33

-

3.82E-07

Lipid, fatty acid and steroid metabolism

879

74

36.87

+

8.63E-07

B-cell- and antibody-mediated immunity

503

3

21.1

-

1.58E-04

Pheromone response

303

0

12.71

-

4.10E-04

Nucleoside, nucleotide and nucleic acid metabolism

3851

213

161.55

+

5.50E-04

Cell surface receptor mediated signal transduction

2601

69

109.11

-

1.82E-03

Carbohydrate metabolism

608

47

25.51

+

2.24E-03

Cell motility

356

33

14.93

+

4.71E-03

Other metabolism

627

46

26.3

+

8.36E-03

Intracellular protein traffic

1044

68

43.8

+

1.02E-02

Fatty acid beta-oxidation

24

7

1.01

+

1.70E-02

Heart development

52

10

2.18

+

1.82E-02

Cell structure and motility

1128

70

47.32

+

2.99E-02

Protein targeting and localization

217

20

9.1

+

3.57E-02

Phospholipid metabolism

148

17

6.21

+

3.58E-02

Stress response

222

22

9.31

+

3.77E-02

Polyphosphate biosynthesis

3

3

0.13

+

4.37E-02

Mitosis

374

31

15.69

+

5.47E-02

Protein metabolism and modification

3819

195

160.2

+

7.21E-02

Amino acid metabolism

248

21

10.4

+

7.45E-02

Sulfur redox metabolism

24

6

1.01

+

8.85E-02

General vesicle transport

270

24

11.33

+

9.39E-02
